# Decontamination and Remediation of the Sulfur Mustard Simulant CEES with “Off‐the‐Shelf” Reagents in Solution and Gel States: A Proof‐of‐Concept Study

**DOI:** 10.1002/open.201700063

**Published:** 2017-06-05

**Authors:** Jennifer R. Hiscock, Gianluca P. Bustone, Ewan R. Clark

**Affiliations:** ^1^ School of Physical Sciences University of Kent Park Wood Road Canterbury Kent CT2 7NH UK

**Keywords:** catalysis, chemical warfare agents, hydrogen bonding, mustard agents, supramolecular gels

## Abstract

The decontamination and remediation of sulfur mustard chemical warfare agents remains an ongoing challenge. Herein, we report the use of “off‐the‐shelf” metal salts alongside commercially available peroxides to catalyze the degradation of the simulant 2‐chloroethyl ethyl sulfide (CEES) in solution and encapsulated within a supramolecular gel.

Sulfur mustards, particularly bis(2‐chloroethyl)sulfide (HD), are a well‐documented class of chemical warfare agent (CWA).^1^ They act as vesicants, reacting through the cyclic intermediate shown in Scheme 1, 2 with biological macromolecules such as DNA.^3^, ^4^, ^5^, ^6^ Furthermore, there are currently no medical countermeasures available to treat the basic cause of a mustard agent injury.^7^

**Scheme 1:**
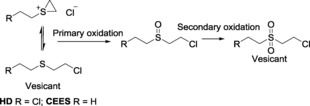
The detoxification of HD through primary oxidation to the sulfoxide followed by secondary oxidation to the sulfone.

Despite control of these substances through the Geneva Protocol (1925)^8^ and Chemical Weapons Convention (1993),^9^ HD use continues.^6^, ^10^, ^11^, ^12^ Therefore, the development of cheap and accessible decontamination and remediation technologies is of great importance, with design informed through fundamental studies, which explore the evaporation, degradation, and vapor emission properties of HD under environmental conditions, such as those recently reported by Jong et al.^13^ Efforts in this area are underrepresented for this class of CWA in comparison to others, as illustrated by Churchill and co‐workers in an extensive Review of CWA destruction and detection methods.^14^

The catalytic detoxification of HD, as overviewed by Smith, is achieved through three main reactive processes: oxidation, dehydrohalogenation, and hydrolysis.^15^ Here, in this proof‐of‐concept study, we report the use of readily obtainable, cheap materials for the remediation of the HD simulant 2‐chloroethyl ethyl sulfide (CEES) through catalytic oxidation of the central sulfur functionality, which prevents the formation of the reactive cyclic cationic intermediate. Over oxidation to produce the sulfone is not desirable, as this compound is also known to act as a vesicant. Metal‐catalyzed formation of chiral sulfoxides is well studied, but achiral catalytic oxidation has largely been ignored—in the laboratory, racemic oxidation is easily accessed at elevated temperatures with simple peroxides.^16^ Oxidative remediation of HD “in the field”, in contrast, requires minimal solvent use, ambient reaction conditions, and reasonable reaction rates to be useful, but not chiral control.

Metal acetylacetonates have frequently been employed as soluble metal sources for the in situ formation of chiral sulfur oxidation catalysts,^17^, ^18^ and it has been observed that preformed catalysts give better *ee* values, which can be attributed to catalytic oxidation owing to remaining M(acac)_2_.^19^ In light of this, and the known utility of metal acetylacetonates as oxidation catalysts in other systems,^20^ we screened a range of first row transition metal acetylacetonate complexes as “off‐the‐shelf” catalysts for the oxidation of HD‐simulant CEES.

Initial studies were conducted in a two‐phase system of CDCl_3_/aqueous H_2_O_2_ (30 wt %) solution, with the reactions monitored by using ^1^H NMR spectrocopy; the results are shown in Figure 1 and Table 1. Mustard agents and their related simulants are highly soluble in organic solvents and the biphasic conditions confine the CWA simulant to an organic phase, sealing it beneath the aqueous hydrogen peroxide solution. This limits CEES transfer through both physical contact and evaporation, with the added benefit that aqueous byproducts or starting materials are easily separated from the organic phase after the neutralization process ends.

**Figure 1:**
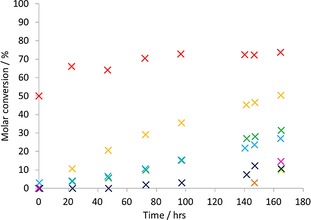
Percentage of CEES consumed under the following conditions: CEES (0.20 mm), catalyst (0.01 mm), CDCl_3_ (1.00 mL), and 30 % hydrogen peroxide in water (0.10 mL) without shaking: **×** VO(acac)_2_; **×** Mn(acac)_3_; **×** Co(acac)_3_; **×** Ni(acac)_2_; **×** Zn(acac)_2_; **×** Cr(acac)_3_; **×** Cu(acac)_2_; **×** Fe(acac)_3_.

**Table 1:** Percentage of CEES oxidized after 165 h under the following conditions: CEES (0.20 mm), catalyst (0.01 mm), CDCl_3_ (1.00 mL), and 30 % hydrogen peroxide in water (0.10 mL) without shaking. Figures given to the nearest whole number.

Catalyst^[a]^	Total oxidation [%]	Primary oxidation [%]	Secondary oxidation [%]
VO(acac)_2_	74	49	24
Mn(acac)_3_^[a]^	3	3	0
Co(acac)_3_	51	46	4
Ni(acac)_2_	10	9	1
Zn(acac)_2_	32	30	1
Cr(acac)_3_	27	26	1
Cu(acac)_2_	11	10	1
Fe(acac)_3_	15	13	2

[a] No data available at 165 h; data obtained after 147 h.

The metal complexes were found to increase the rate of CEES oxidation in the order of VO(acac)_2_ > Co(acac)_3_ > Zn(acac)_2_ > Cr(acac)_3_ > Fe(acac)_3_ > Cu(acac)_2_ Ni(acac)_2_ > Mn(acac)_3_. Although VO(acac)_2_ is inarguably the most efficient oxidation catalyst, it leads to significant overoxidation. In light of the toxicity, cost, low activity, and increased cost of the of manganese, cobalt, nickel, and chromium complexes, as well as the extreme difficulty in monitoring the iron‐containing reaction with NMR, we chose to focus our efforts on the copper‐ and zinc‐based systems; although work is ongoing exploring other systems.

Copper sulfoxidation catalysis is well known,^21^, ^22^ though general mechanisms are not established. Direct combination of Cu(acac)_2_ and CEES in CDCl_3_ leads to paramagnetic broadening of the protons of the S‐adjacent methylene groups, indicating interaction of the sulfur with the copper center. Hypothesizing that this complexation may play a role in the overall oxidation process, we synthesized the more Lewis acidic Cu(hfac)_2_⋅H_2_O, which showed analogous binding behavior with CEES (Figure 2), also observed by electron paramagnetic resonance (see the Supporting Information).

**Figure 2:**
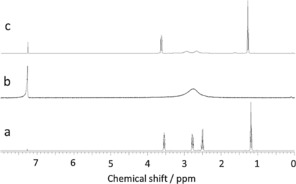
^1^H NMR spectra of a) CEES (0.20 mm), b) Cu(hfac)_2_⋅H_2_O (0.01 mm), c) Cu(hfac)_2_⋅H_2_O (0.01 mm) and CEES (0.20 mm) in CDCl_3_ (1 mL).

Comparative studies of Cu(acac)_2_ and Cu(hfac)_2_⋅H_2_O were conducted in similar manner to before, but, mindful that diffusion from the aqueous layer might limit oxidation rates, the mixtures were agitated for 2 s after addition.

As shown in Figure 3, both Cu^II^ complexes at 5 mol % with respect to CEES result in the increased oxidation rate of this simulant. Over a 125 h period, 28, 43, and 66 % of the CEES was oxidized without catalyst, with Cu(acac)_2_, and with Cu(hfac)_2_⋅H_2_O, respectively. It is tentatively proposed that the enhanced Lewis acidity of Cu(hfac)_2_⋅H_2_O, owing to the electron‐withdrawing CF_3_ groups of the hfac ligand, strengthens substrate binding and subsequent activation. The slight agitation at the beginning of the experimental protocol and hfac incorporation gives rates for this Cu^II^ compound competitive with those shown in Figure 1.

**Figure 3:**
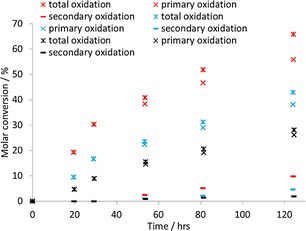
Percentage of CEES oxidized under the following conditions: CEES (0.20 mm), catalyst (0.01 mm), CDCl_3_ (1.00 mL) and 30 % hydrogen peroxide in water (0.10 mL) with shaking: × Cu(hfac)_2_⋅H_2_O; × Cu(acac)_2_; × no catalyst.

There are many instances where the neutralization of mustard agents in the solution state is undesirable. Liquids may flow, adhere to surfaces, and be easily vaporized. Any neutralization process that occurs unconfined still represents a relatively high contamination hazard. However, these associated risks can be minimized by confining the solution‐state neutralization processes within a solid. The body of work produced by Gale and co‐workers has explored the use of supramolecular gels as decontamination and remediation materials for organophosphorus (OP) CWAs.^23^, ^24^, ^25^, ^26^, ^27^ These materials were found to undergo physical‐state transitions in the presence of the OP CWA soman or simulants, showing potential use as OP CWA sensors. They were also shown to absorb OP CWA simulants, destroying them in situ, and prompting interest in their use as possible decontamination materials. Here, we present a complimentary system, presenting supramolecular gels as remediation and decontamination materials for HD, in combination with peroxides and metallocatalysts.


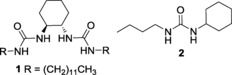


Supramolecular gelators self‐associate through the formation of intermolecular hydrogen bonds, resulting in the creation of a fibrous network (Figure 4) trapping the remaining sol to produce a gel. These gels are sensitive to external stimuli, undergoing gel–sol or sol–gel transitions as desired. We explored a proof‐of‐principle system using gelator **1** 28 in combination with Cu(hfac)_2_ for the in situ remediation of CEES within a gel. Attempts to incorporate Zn(acac)_2_ into a gel were prevented by poor solubility under gelating conditions. Compound **2** was used to test the reactivity of the urea functionality of **1** towards the other components of the remediatory gel system in solution. The minimum concentration of **1** needed to gelate a chloroform solution saturated with Cu(hfac)_2_⋅H_2_O was identified as 25 mg mL^−1^ through a series of inversion tests.

**Figure 4:**
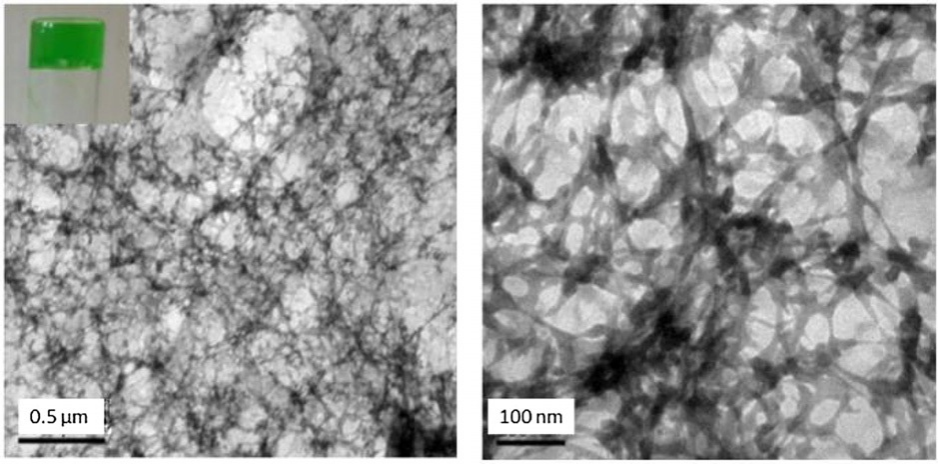
TEM images of a xerogel obtained from **1** (35 mg) and a saturated solution of Cu(hfac)_2_⋅H_2_O in CHCl_3_ (1 mL)_,_ illustrating supramolecular fiber formation.

We were unable to use aqueous H_2_O_2_ for these gel studies, as mass transport between the gel and aqueous phase is incredibly slow. Instead, we incorporated a 5–6 m solution of *tert‐butyl* peroxide (*t*BuOOH) in decane into our gel systems to aid the oxidation process. This peroxide is commercially available on a bulk scale at a reasonable cost.

These proof‐of‐concept gel studies were monitored by using ^1^H NMR spectroscopy, with the conversion calculated through comparative integration of these spectra over time, as shown Figure 5. Owing to peak broadening effects, we were unable to differentiate between primary and secondary CEES oxidation, although evidence from these spectra suggests that both of these oxidative processes are occurring within these samples. The encapsulation of CEES (0.25 mm) and *t*BuOOH (0.25–0.30 mm) in the presence of 4 mol % Cu(hfac)_2_⋅H_2_O, with respect to CEES, was found to increase the percentage of oxidation product from 13 to 50 % over 27 h, as compared to a sample without catalyst. Further gel/sol studies conducted with varying concentrations of gelator, peroxide, and catalyst (see the Supporting Information) exhibit minimal peak broadening and suggest only the presence of primary oxidative processes, meaning the presence of the undesired sulfone is not observed under these conditions.

**Figure 5:**
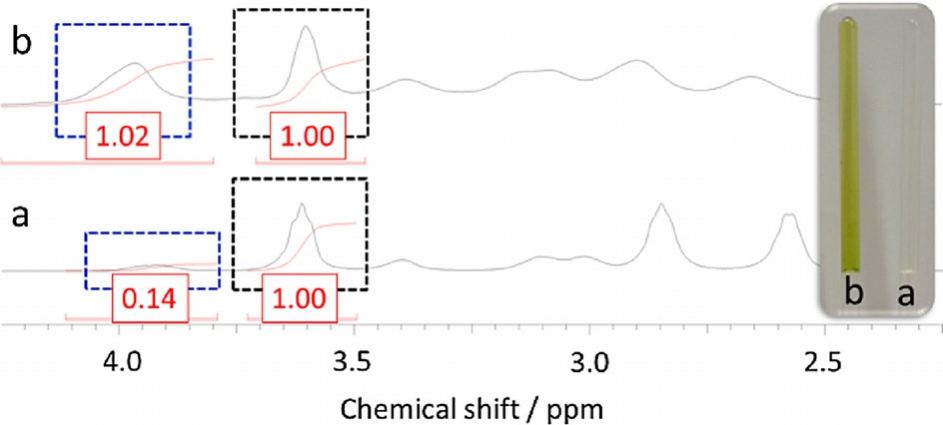
^1^H NMR spectra after 27 h of a) **1** (40 mg), simulant (0.25 mm), *t*BuOOH (0.25–0.30 mm) in CDCl_3_ (0.92 mL); b) **1** (40 mg), simulant (0.25 mm), Cu(hfac)_2_⋅H_2_O (0.01 mm), *t*BuOOH (0.25–0.30 mm) in CDCl_3_ (0.92 mL). Black: resonance belonging to CEES; blue: resonance belonging to the corresponding oxidized products.

In summary, this proof‐of‐concept study has shown that the cheap, commercially available transition‐metal complexes are able to act as catalysts for the oxidation of the sulfur mustard simulant CEES in a two‐phase solution with hydrogen peroxide. We have shown that limited agitation and substitution of acac ligand for hfac enhances the activity of the Cu^II^‐based catalysts. These studies are the first example of the use of Cu(acac)_2_ derivatives as sulfoxidation catalysts rather than pre‐catalysts. Finally, Cu(hfac)_2_⋅H_2_O can be incorporated into a supramolecular gel to enable the catalysis of CEES oxidation within the solid state in the presence of the organic oxidant *t*BuOOH. Work is ongoing to fully understand this catalytic process under a variety of environmental conditions, determine effective CWA remediation times, and investigate further combinations of catalyst, solvent, and peroxide. Next‐generation systems are also under investigation to limit the oxidation process to the sulfoxide only, preventing further production of vesicants.

## Conflict of interest

*The authors declare no conflict of interest*.

## Supporting information

As a service to our authors and readers, this journal provides supporting information supplied by the authors. Such materials are peer reviewed and may be re‐organized for online delivery, but are not copy‐edited or typeset. Technical support issues arising from supporting information (other than missing files) should be addressed to the authors.

Supplementary
