# Supplementary material for: Decontamination and Remediation of the Sulfur Mustard Simulant CEES with “Off‐the‐Shelf” Reagents in Solution and Gel States: A Proof‐of‐Concept Study
Source: ChemistryOpen. 2017 Jun 5;6(4):497–500. doi: 10.1002/open.201700063 (PMC5542751; doi:10.1002/open.201700063)
Supplement: Supplementary file 1 — Supplementary [file OPEN-6-497-s001.pdf]

Supporting Information

© 2017 The Authors. Published by Wiley-VCH Verlag GmbH & Co. KGaA, Weinheim

Decontamination and Remediation of the Sulfur Mustard Simulant CEES with "Off-the-Shelf" Reagents in Solution and Gel States: A Proof-of-Concept Study

Jennifer R. Hiscock,* Gianluca P. Bustone, and Ewan R. Clark*[a]

open_201700063_sm_miscellaneous_information.pdf

Supporting Information

Table of Contents

Experimental 2

Characterisation NMR 3

EPR studies 4

Coordination studies 6

Oxidative breakdown of CEES 7

- Primary Solution state studies 7
- Secondary Solution state studies 18
- Solid state studies 21

Gel formation 24

Sol and Gelator simulant studies 24

References 27

Experimental

General remarks: All reactions were performed under slight positive pressure of nitrogen using oven dried glassware. NMR spectra were determined on a Jeol ECS-400 spectrometer with the chemical shifts reported in parts per million (ppm), calibrated to the centre of the solvent peak set. All solvents and starting materials were purchased from chemical stores where available. Melting points were recorded in open capillaries on a Stuart SMP10 melting point apparatus and are uncorrected. Infrared (IR) spectra were recorded on a Shimadzu IR-Affinity 1, and reported in wavenumbers (cm^{-1}). EPR Studies were performed using an ADANI CMS 8400 X-Band spectrometer at room temperature, on 10μl samples within quartz tubes. Solution state spectra were simulated using the EasySpin[1] toolbox for MatLab using the *garlic* core modelling function. Linewidths were allowed to freely refine to a mixed Gaussian/Lorenzian lineshape. All spectra were recorded with Modulation Amplitude of 200 mT and a scan rate of 1 mTs^{-1}. For primary solution state studies metal salts were purchased from commercial sources, for secondary solution state studies the metal salts were synthesised in house to test for ease of synthesis.

Synthesis of $Cu(acac)_2$: As described published by Maverick and co-workers.[2]

Synthesis of $Cu(hfac)_2.H_2O$: As described published by Glidewell.[3]

Synthesis of 1: Dodecylamine (0.25 g, 1.35 mmol) was added to a stirring solution of carbonyl diimidazole (CDI) (0.26 g, 1.62 mmol) for two hours. After this time (1*S*,2*S*)-cyclohexane-1,2-diamine (0.07 g, 0.68 mmol) was added and the solution heated to 60 °C overnight. The reaction mixture was then washed with water (50 mL) and the resultant solid removed by filtration, titrated in ether (20 mL), followed by methanol (40 mL) and finally water (40 mL). The remaining white solid was removed by filtration and dried under reduced pressure. Yield 53 % (0.18 g, 0.36 mmol). NMR spectra were found to match those previously reported by Feringa and co-workers.[4]

Synthesis of 2: Butylisocyanate (0.19 g, 1.96 mmol) was added to a stirring solution of cyclohexylamine (0.19 g, 1.96 mmol) in chloroform (10 mL) for three hours. The mixture was taken to dryness and the resulting oil dissolved in hexane (5 mL). The product precipitated as a white solid and was isolated by filtration. Yield 24 % (0.096 g, 0.46 mmol). NMR spectra were found to match those previously reported by King and co-workers.[5]

Characterisation NMR

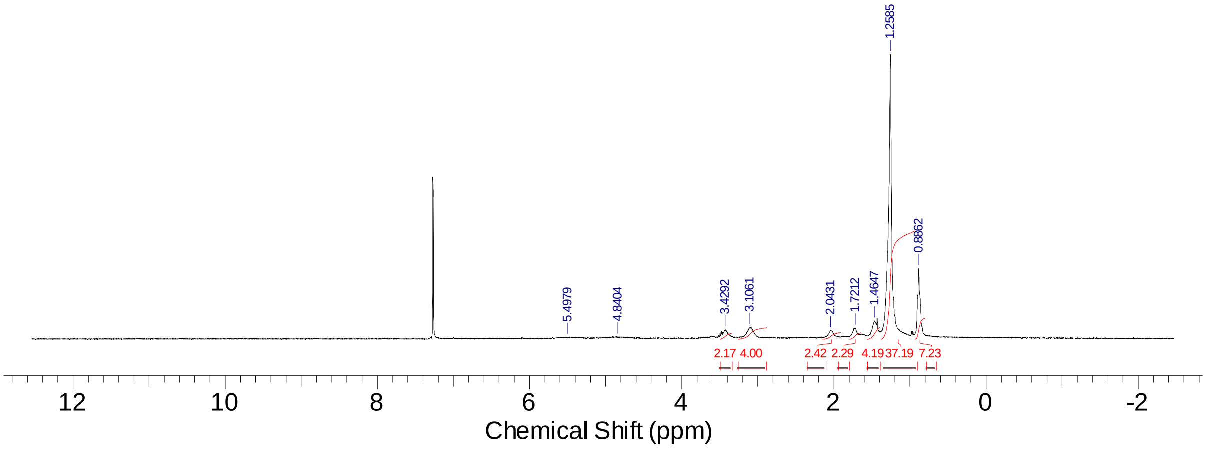

Figure S1 ^{1}H NMR of compound **1** in $CDCl_3$ at approximately 60 °C. Position of NH resonances differs slightly from those previously reported. We believe this is due to the concentration of the sample and the corresponding self-associative properties of the molecule combined with peak broadening effects.

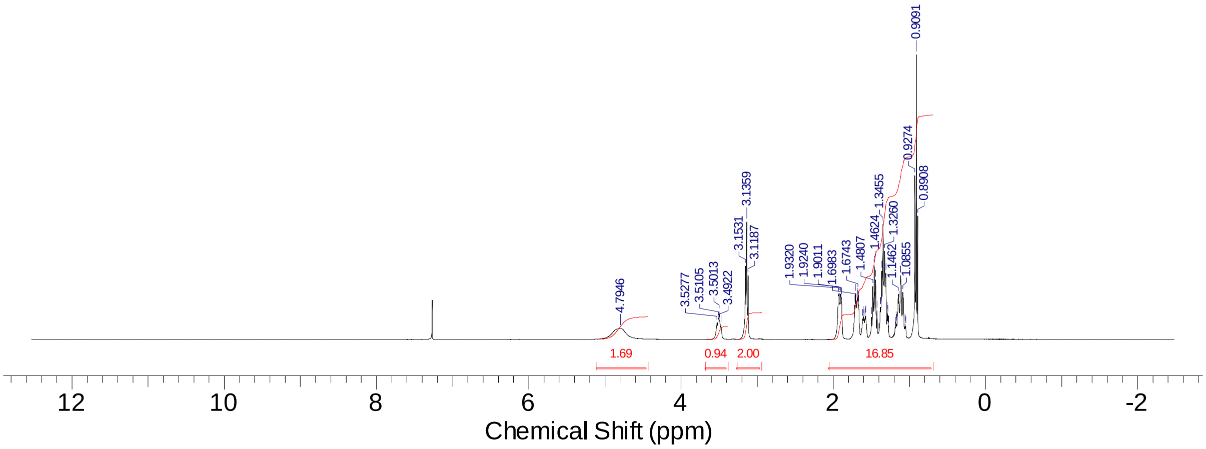

Figure S2 ^{1}H NMR of compound **2** in $CDCl_3$ at approximately 60 °C.

EPR studies

Experimental Method: EPR samples were made up with laboratory analytical grade chloroform and no special measures were used to exclude air or moisture to maintain conditions comparable to those of the reaction. $Cu(hfac)_2.H_2O$ (50 mg, 0.1 mmol) was stirred with gently warmed chloroform (1 cm^3) to give a saturated stock solution (Solution A) at 0.1M concentration. Likewise, CEES (12 µl, 0.1 mmol) was dissolved in chloroform (1 cm^3) to form a stock solution (Solution B) at 0.1M concentration. These stock solutions were then used to make up solutions of $Cu(hfac)_2$ with varying concentrations ratio of copper to CEES to attempt to drive the equilibrium towards domination by $Cu(hfac)_2$.CEES. For Samples 1 to 3, the $Cu(hfac)_2$ concentration was kept approximately constant for ease of comparison.

Table S1 Constituents of samples 1-4.

Sample Name	$Cu(hfac)_2.H_2O$ Added	CEES Added	$CHCl_3$ added
1	100 µl Solution A	-	900 µl $CHCl_3$
2	100 µl Solution A	100 µl Solution B	800 µl $CHCl_3$
3	100 µl Solution A	12 µl neat CEES	900 µl $CHCl_3$
4	100 µl Solution A	100 µl neat CEES	-

Results and Discussion: Attempts to crystalise the presumed adduct of $Cu(hfac)_2$.CEES were unsuccessful, yielding only crystalline samples of $Cu(hfac)_2.H_2O$. This is presumably due to the competition of H_2O and CEES to copper, coupled with the volatility of CEES in the cases of attempted crystalisation by slow evaporation.

The EPR spectral parameters for $Cu(hfac)_2.H_2O$ are in good agreement with literature values.[6, 7] Upon addition of increasing quantities of CEES, there is a gradual increase in g_{iso} value and concomitant decrease in the A_{iso} coupling to the copper nucleus as the concentration of CEES increases. This is accompanied by an increase in asymmetric line broadening, indicating a movement into the fast-motional regime.

In the absence of good solid state data for the complex to properly model rotational anisotropy, the values for Sample 4 should be regarded as indicative only. The EPR spectra were largely similar in appearance, and the spectra and simulations for Samples 1 and 4 are shown as exemplars. It is also plausible that the change in lineshapes relates to the formation of an higher order adduct e.g. $Cu(hfac)_2.(CEES)_2$, but given the significant linewidths involved, there are insufficient data to attempt to model that.

Table S2 Results from EPR studies. Literature Values: g=2.135 A = 67.0 (x10^4 cm-1) = 200 MHz[6]; g=2.133, A = 69(x10^4 cm-1) = 208 MHz[7].

Sample Name	g_{iso}	A_{iso} / MHz	Gaussian , Lorenzian Linewidths / MHz
1	2.128	201.6	3.8, 1.3
2	2.129	206.3	0, 3.4
3	2.131	196.14	2.5, 3.2
4	2.144	163.4	0.6, 3.5

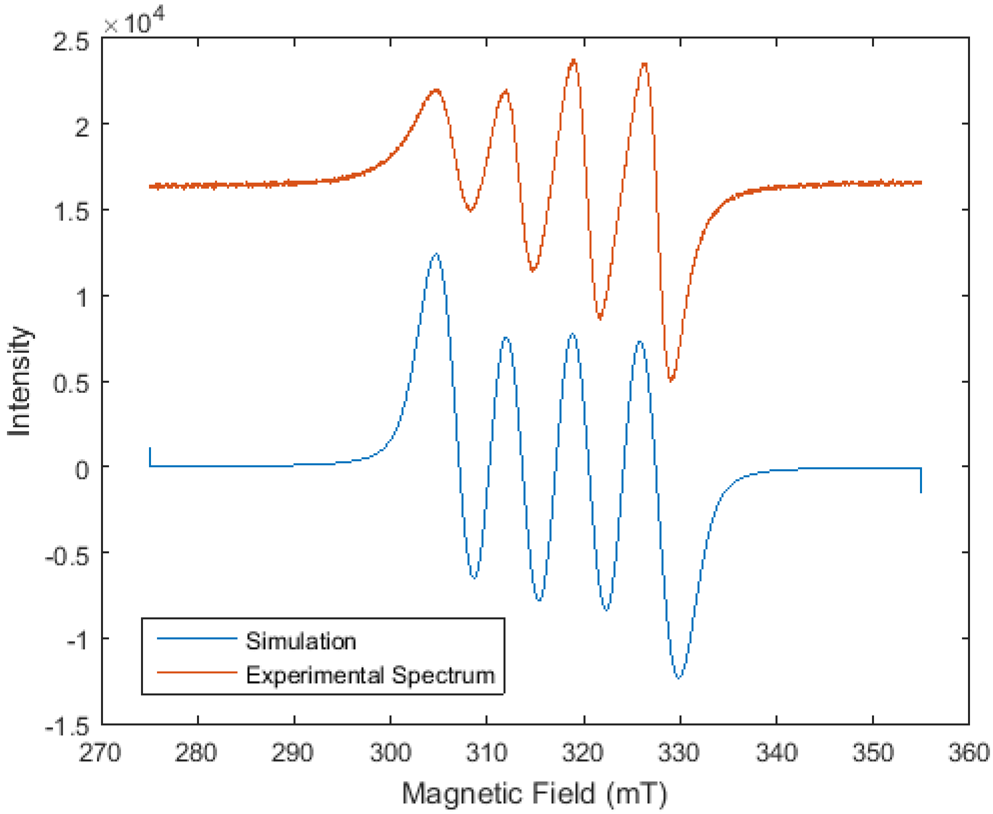

Figure S3 Simulated and Experimental Spectrum for Sample 1.

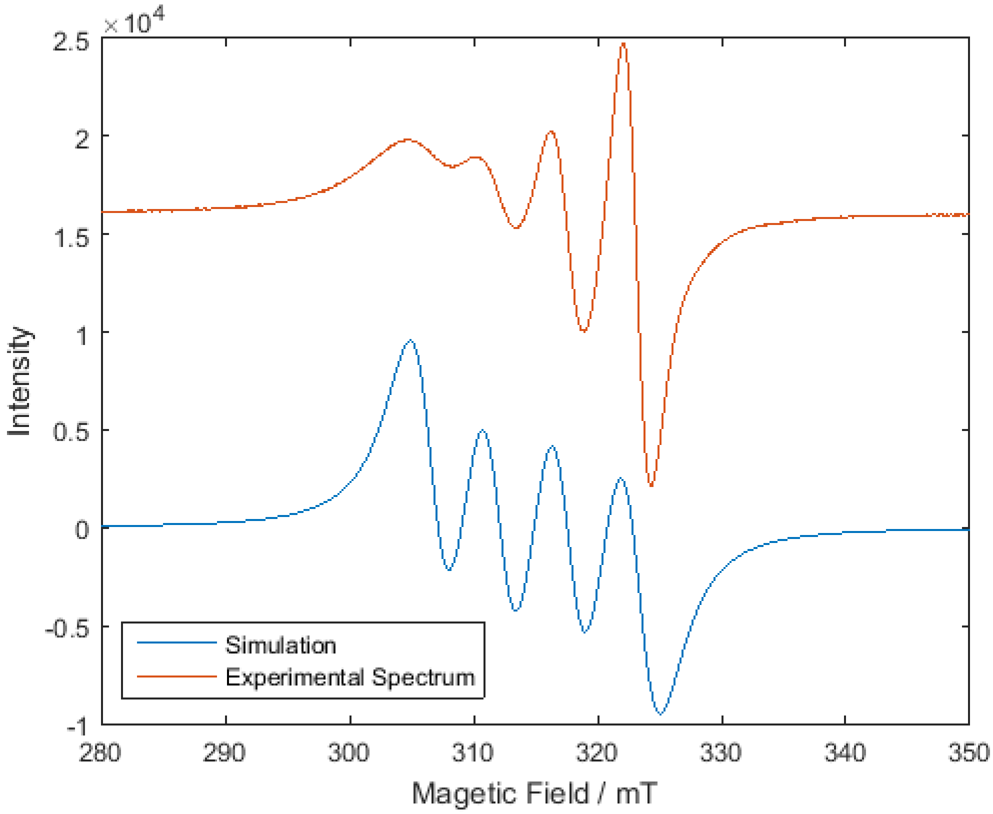

Figure S4 Simulated and Experimental Spectrum for Sample 4.

Coordination studies

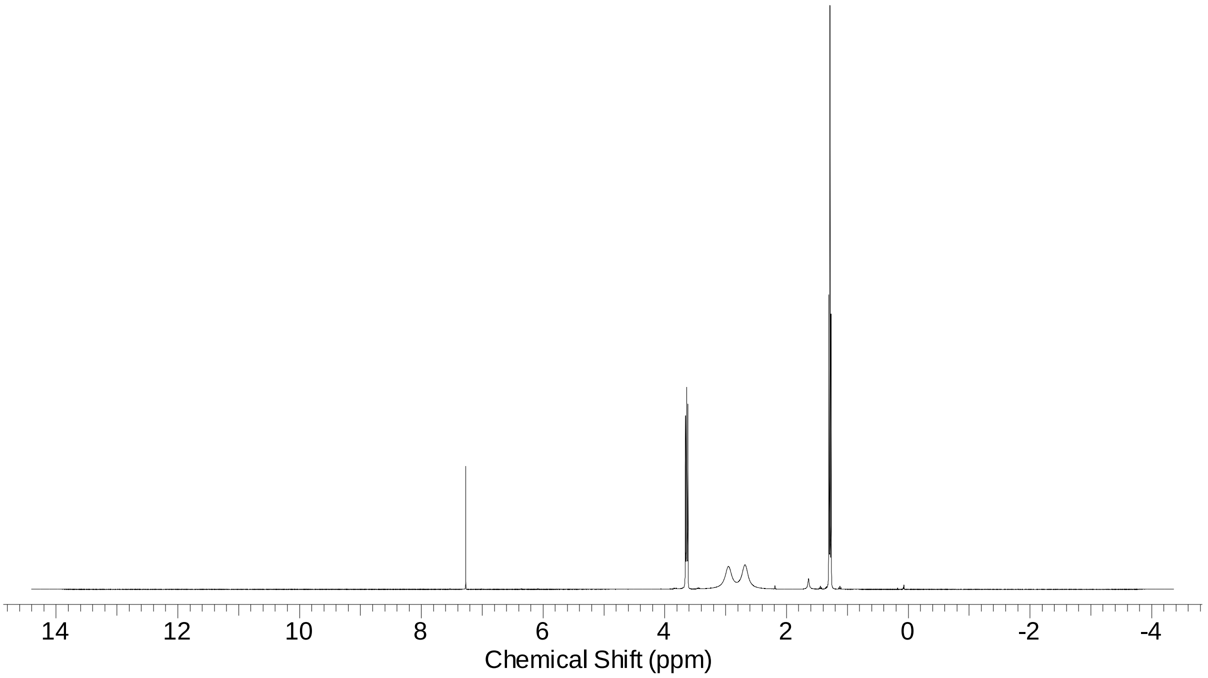

Figure S5 ^{1}H NMR spectrum of $Cu(hfac)_2.H_2O$ (0.01 mM) and CEES (0.20 mM) in $CDCl_3$ (1 mL).

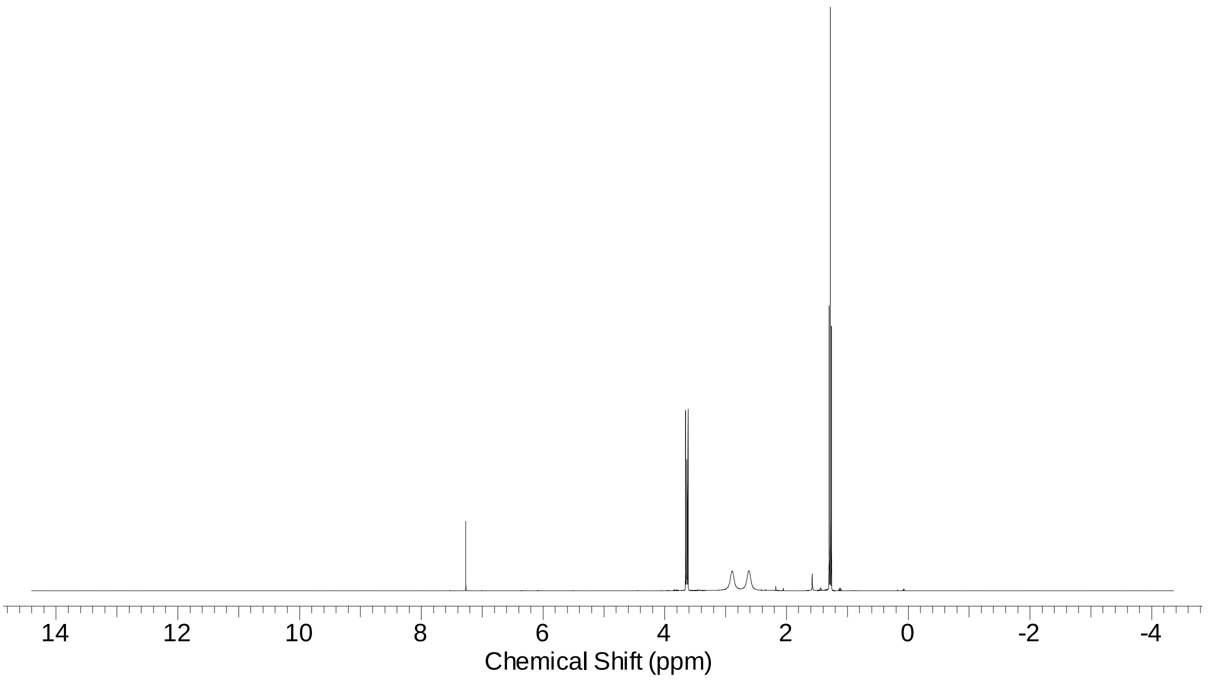

Figure S6 ^{1}H NMR spectrum of $Cu(acac)_2.H_2O$ (0.01 mM) and CEES (0.20 mM) in $CDCl_3$ (1 mL).

Oxidative breakdown of CEES

Primary Solution state studies

Experimental Method: Samples were prepared as detailed in Table S3 in a single NMR tube per experiment, with the hydrogen peroxide layered on top of the $CDCl_3$ mixture containing catalyst and/or simulant as appropriate. The addition of the peroxide was taken as time = 0, at this point it was assumed that no oxidation products were present as ^1H NMR of the stock CEES in $CDCl_3$ showed no breakdown products to be present. The samples were then sealed and disturbed as little as possible for the course of the experiment with the temperature of the samples maintained at 18 ± 2 °C. All experiments were monitored by ^1H NMR. The percentage conversion of simulant to oxidised species was calculated through comparative integration of simulant and product peaks, the results of which are given in Table S3. The initial and final ^1H NMR spectra for each experiment are shown in Figures S7-S30. The identification of the corresponding sulfoxide[8] and sulfone[9] was achieved through comparison with previously published literature values. The sparsity of data points for the Mn, Fe and Ni catalysis tests are due to the paramagnetic broadening which precluded meaningful integration.

Table S3 Constituents of samples 5-20. ^{a} – 30 wt% solution in water.

Sample number	Catalyst	Conc. (mM)	Solvent	Amount (mL)	Simulant	Conc. (mM)	Peroxide	Amount (mL)
5		0.01	$CDCl_3$	1.00	CEES	0.20	NA	-
6		0.01	$CDCl_3$	1.00	CEES	0.20	NA	-
7		0.01	$CDCl_3$	1.00	CEES	0.20	NA	-
8		0.01	$CDCl_3$	1.00	CEES	0.20	NA	-
9		0.01	$CDCl_3$	1.00	CEES	0.20	NA	-
10		0.01	$CDCl_3$	1.00	CEES	0.20	NA	-
11		0.01	$CDCl_3$	1.00	CEES	0.20	NA	-
12		0.01	$CDCl_3$	1.00	CEES	0.20	NA	-
13		0.01	$CDCl_3$	1.00	CEES	0.20	Hydrogen peroxide^a	0.1
14		0.01	$CDCl_3$	1.00	CEES	0.20	Hydrogen peroxide^a	0.1
15		0.01	$CDCl_3$	1.00	CEES	0.20	Hydrogen peroxide^a	0.1
16		0.01	$CDCl_3$	1.00	CEES	0.20	Hydrogen peroxide^a	0.1
17		0.01	$CDCl_3$	1.00	CEES	0.20	Hydrogen peroxide^a	0.1
18		0.01	$CDCl_3$	1.00	CEES	0.20	Hydrogen peroxide^a	0.1
19		0.01	$CDCl_3$	1.00	CEES	0.20	Hydrogen peroxide^a	0.1
20		0.01	$CDCl_3$	1.00	CEES	0.20	Hydrogen peroxide^a	0.1

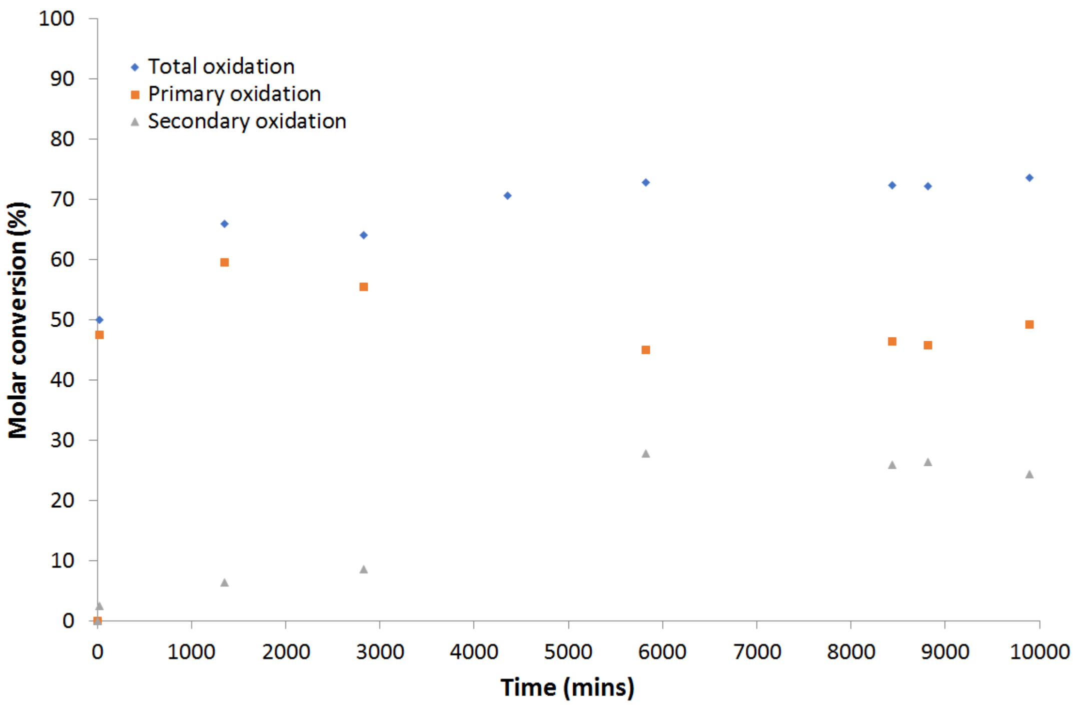

Figure S7 Percentage of CEES oxidised under the following conditions: CEES (0.20 mM), $VO(acac)_2$. (0.01 mM), $CDCl_3$ (1.00 mL) and 30% hydrogen peroxide in water (0.10 mL).

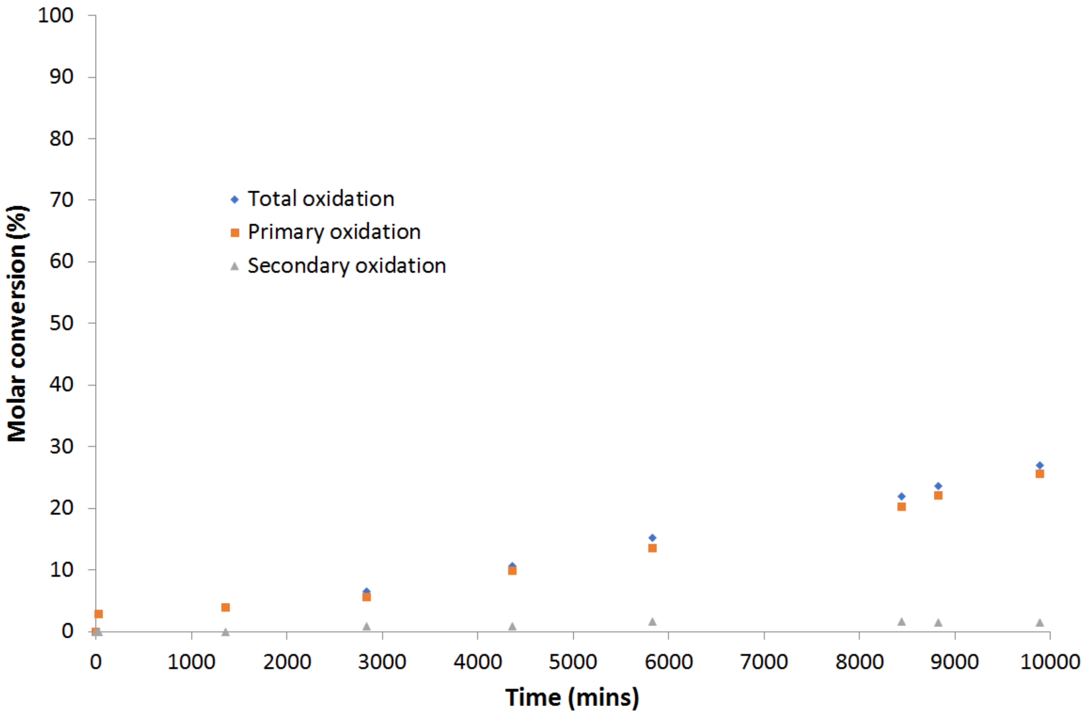

Figure S8 Percentage of CEES oxidised under the following conditions: CEES (0.20 mM), $Cr(acac)_3$. (0.01 mM), $CDCl_3$ (1.00 mL) and 30% hydrogen peroxide in water (0.10 mL).

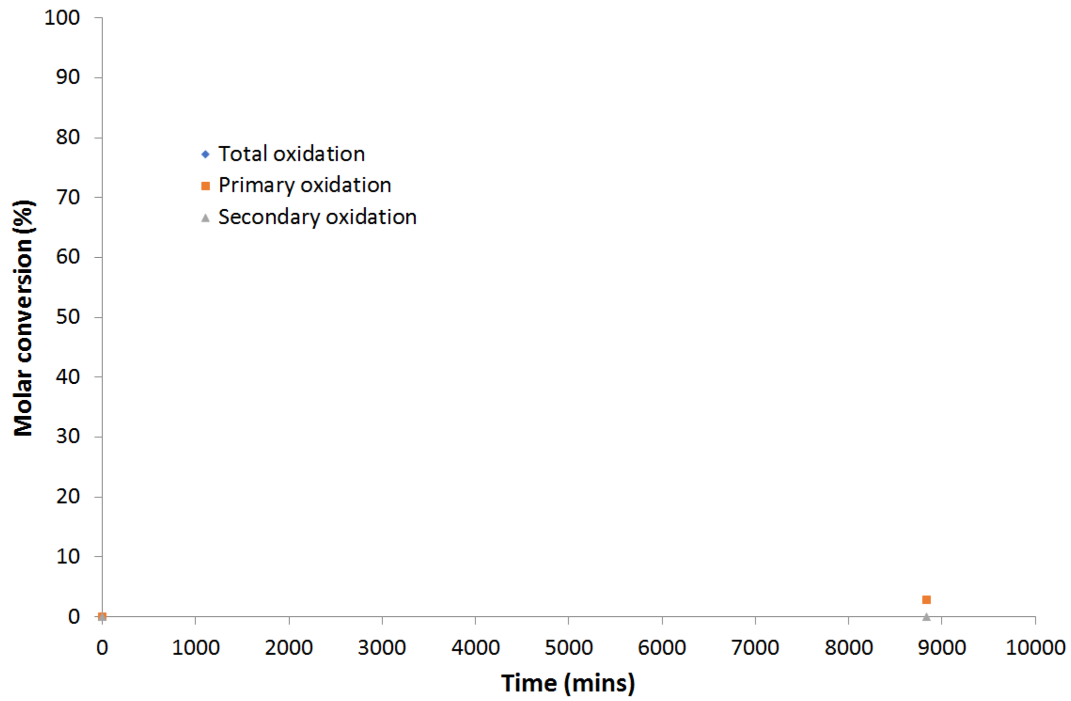

Figure S9 Percentage of CEES oxidised under the following conditions: CEES (0.20 mM), $Mn(acac)_3$. (0.01 mM), $CDCl_3$ (1.00 mL) and 30% hydrogen peroxide in water (0.10 mL).

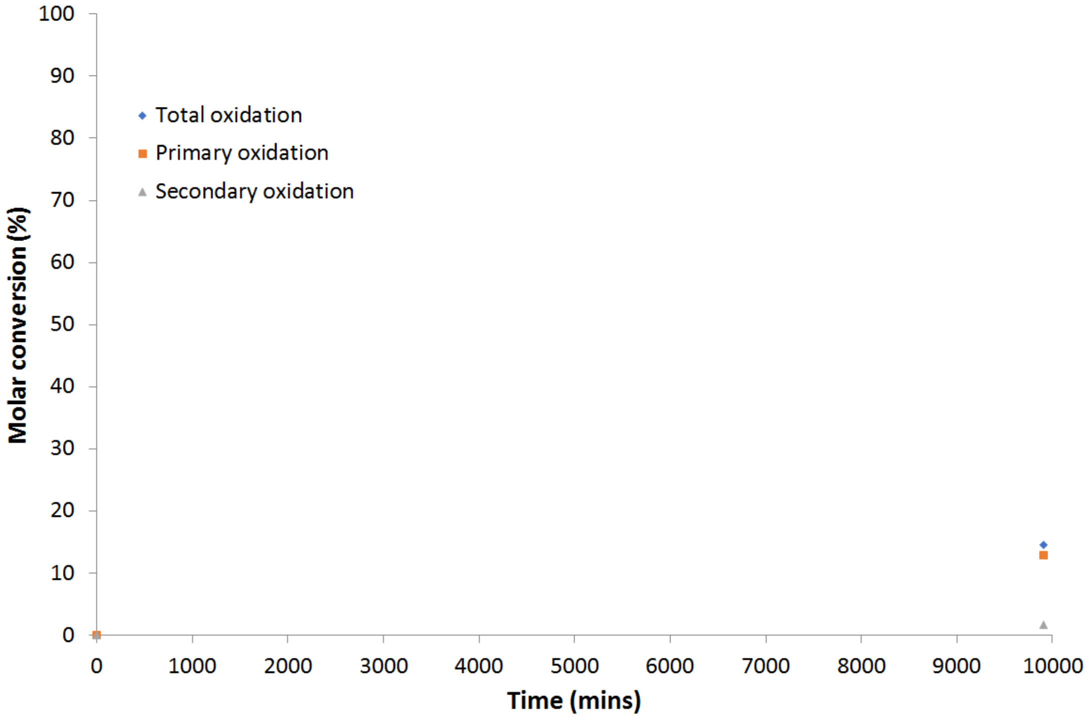

Figure S10 Percentage of CEES oxidised under the following conditions: CEES (0.20 mM), $Fe(acac)_3$. (0.01 mM), $CDCl_3$ (1.00 mL) and 30% hydrogen peroxide in water (0.10 mL).

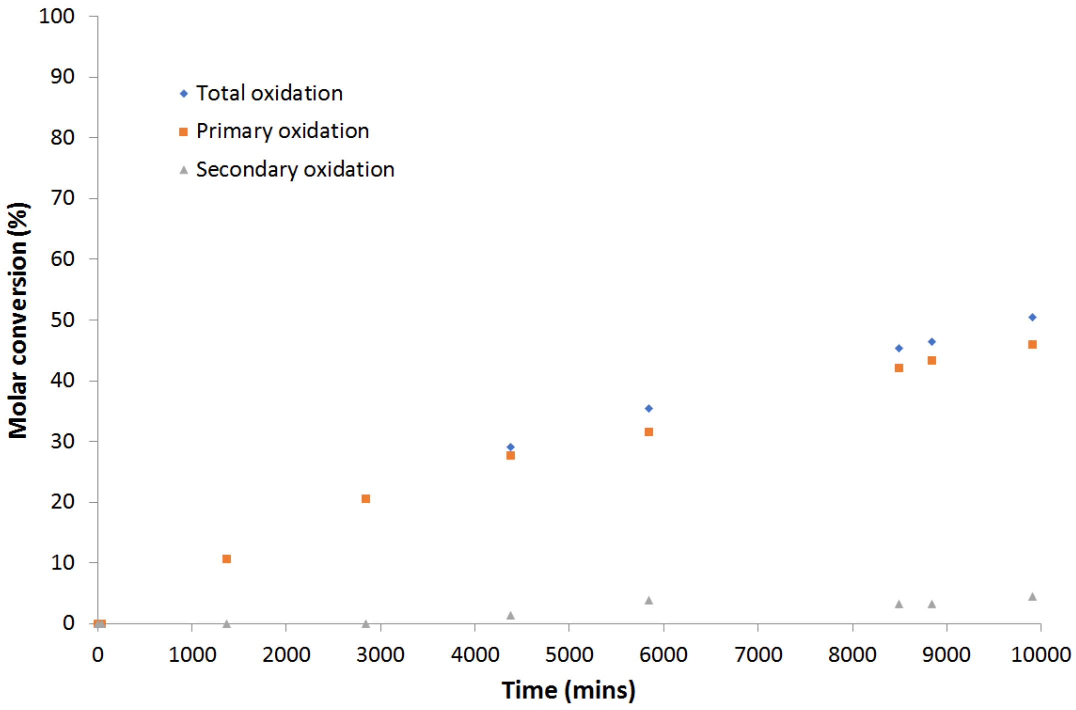

Figure S11 Percentage of CEES oxidised under the following conditions: CEES (0.20 mM), $Co(acac)_3$. (0.01 mM), $CDCl_3$ (1.00 mL) and 30% hydrogen peroxide in water (0.10 mL).

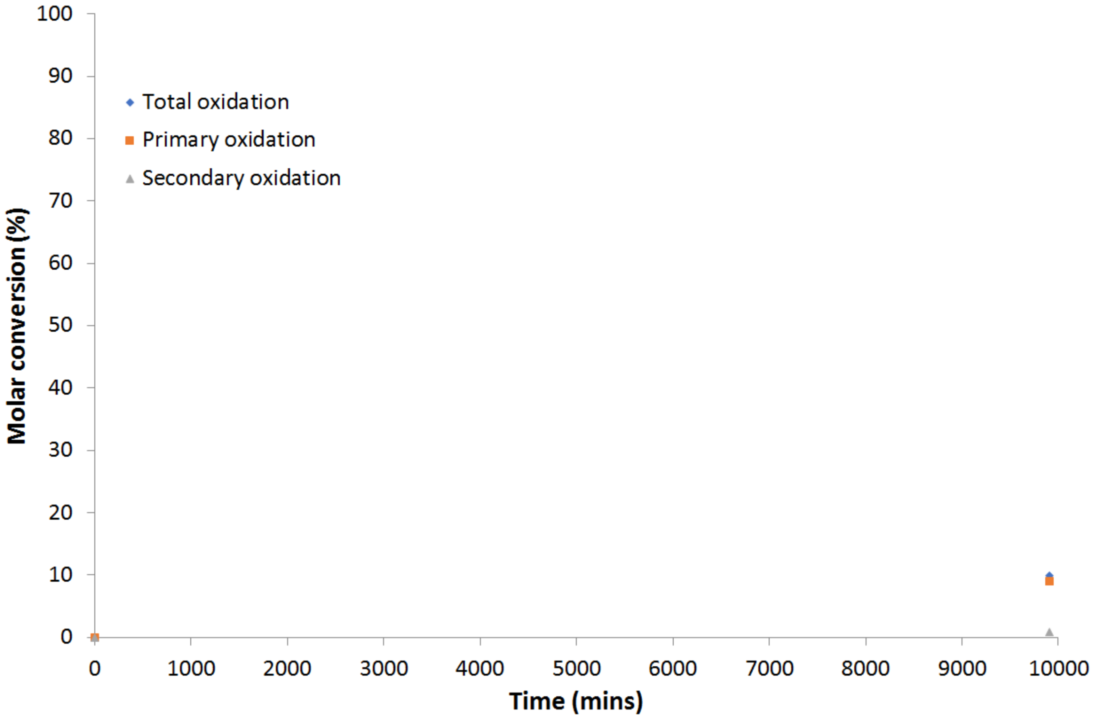

Figure S12 Percentage of CEES oxidised under the following conditions: CEES (0.20 mM), $Ni(acac)_2$. (0.01 mM), $CDCl_3$ (1.00 mL) and 30% hydrogen peroxide in water (0.10 mL).

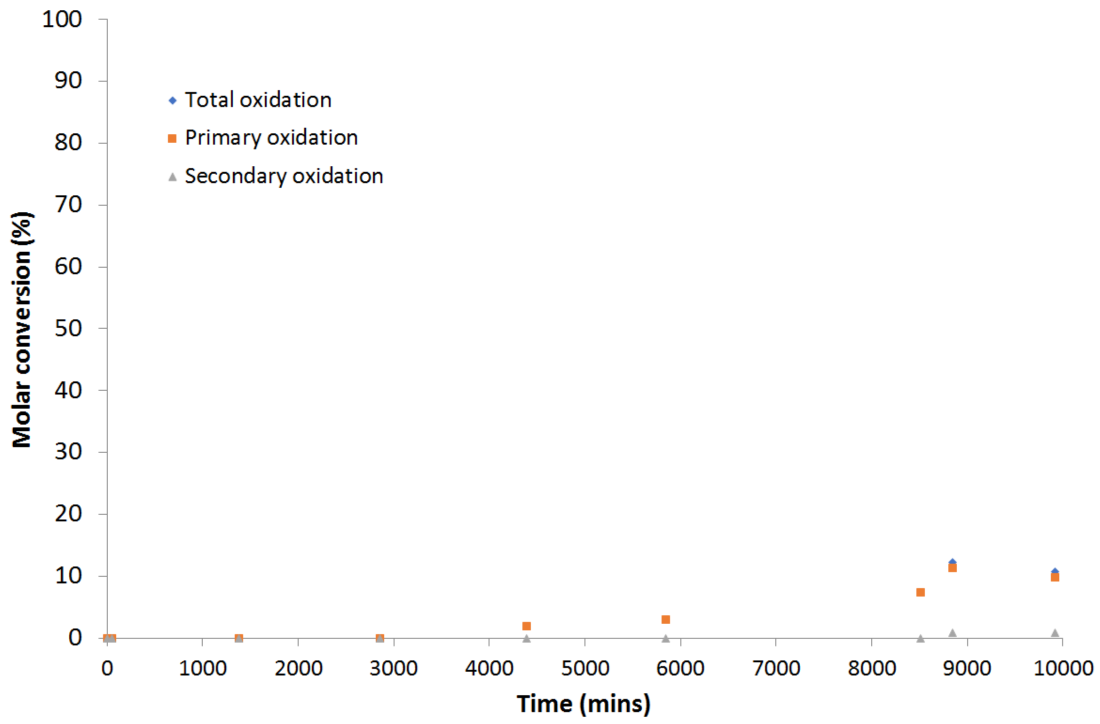

Figure S13 Percentage of CEES oxidised under the following conditions: CEES (0.20 mM), $Cu(acac)_2$. (0.01 mM), $CDCl_3$ (1.00 mL) and 30% hydrogen peroxide in water (0.10 mL).

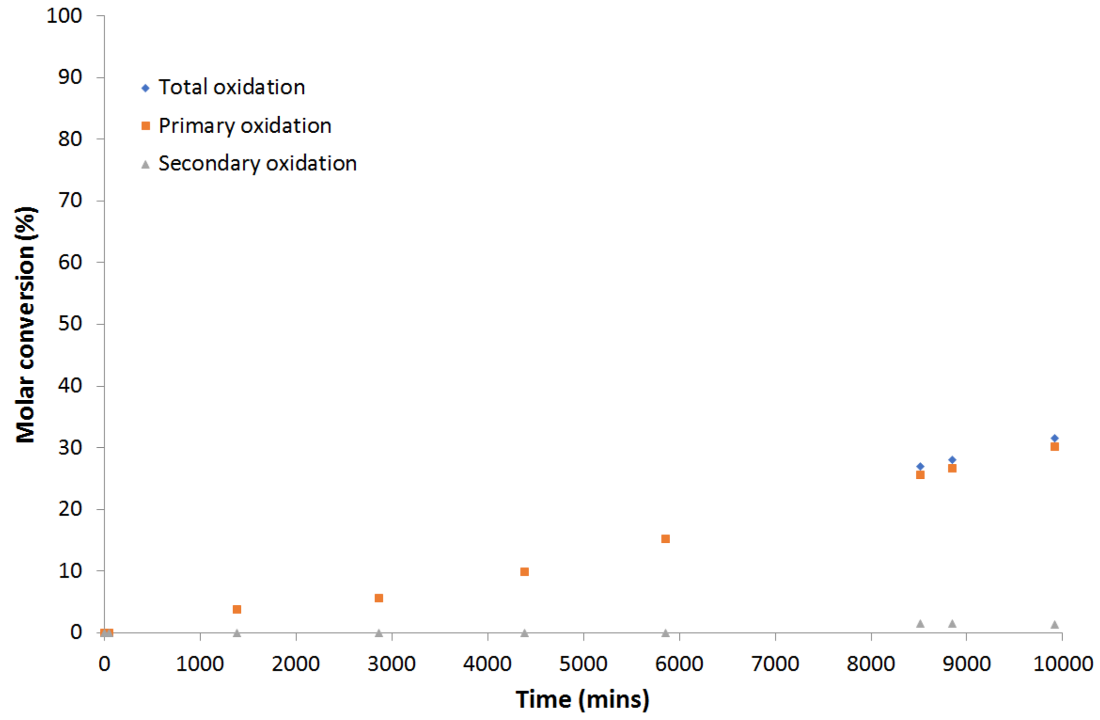

Figure S14 Percentage of CEES oxidised under the following conditions: CEES (0.20 mM), $Zn(acac)_2$. (0.01 mM), $CDCl_3$ (1.00 mL) and 30% hydrogen peroxide in water (0.10 mL).

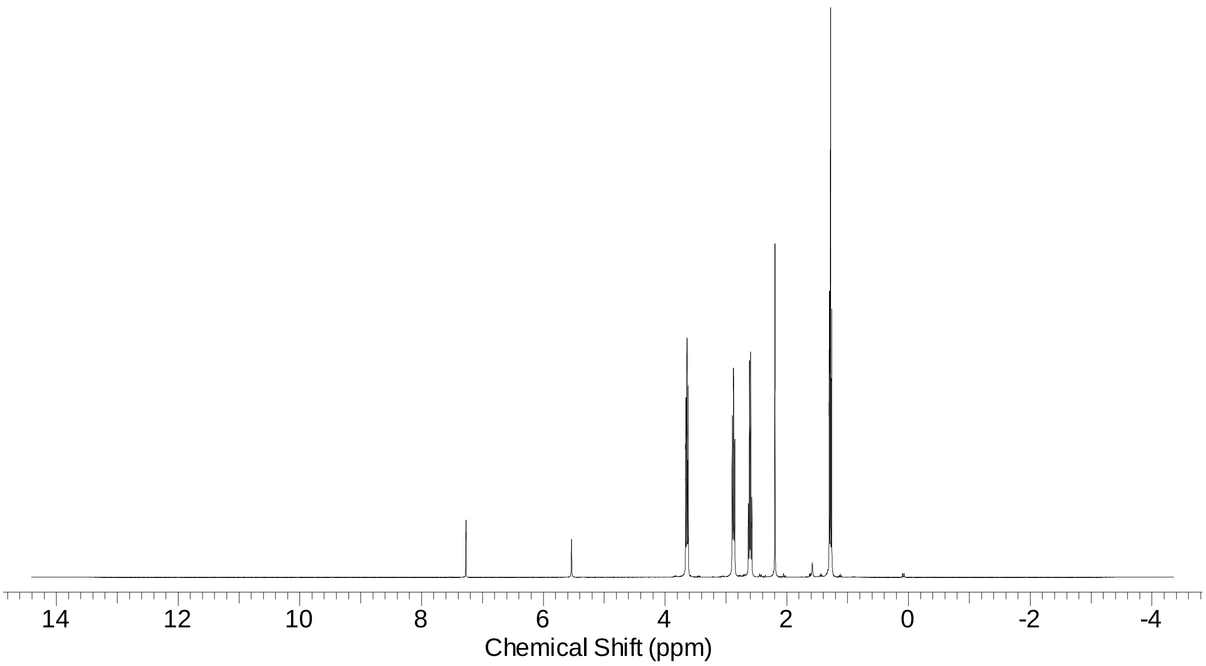

Figure S15 ^{1}H NMR spectrum after 173 hrs of: CEES (0.20 mM), Co(acac)$_3$ (0.01 mM) and $CDCl_3$ (1.00 mL).

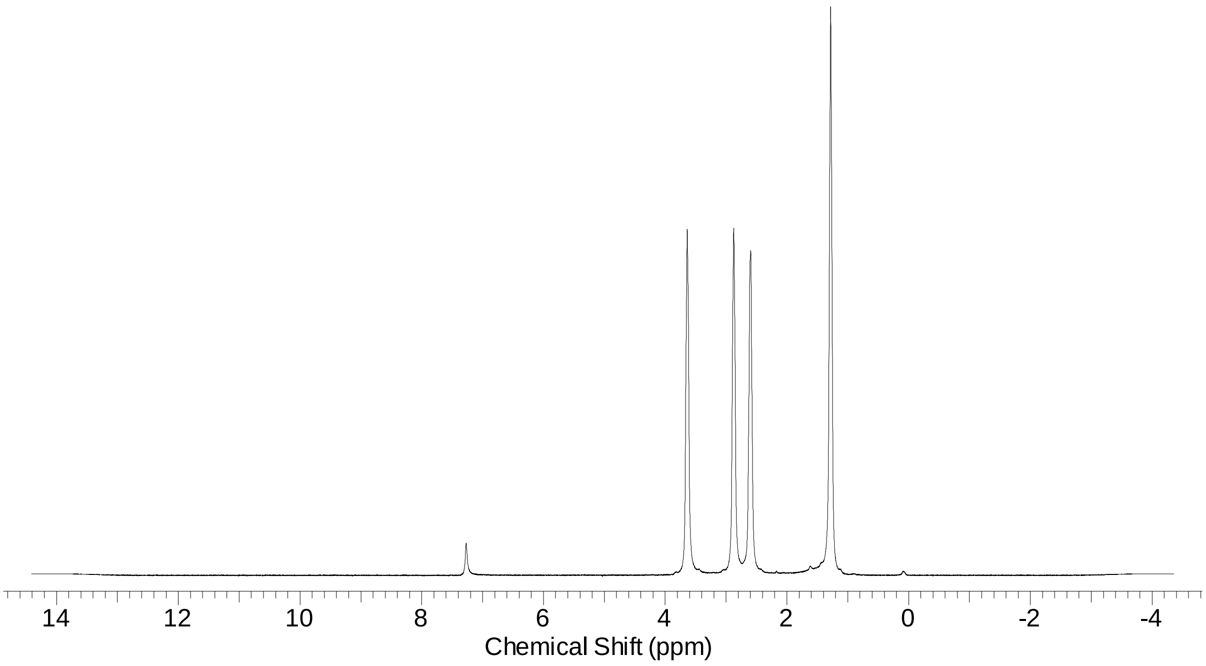

Figure S16 ^{1}H NMR spectrum after 173 hrs of: CEES (0.20 mM), Cr(acac)$_3$ (0.01 mM) and $CDCl_3$ (1.00 mL).

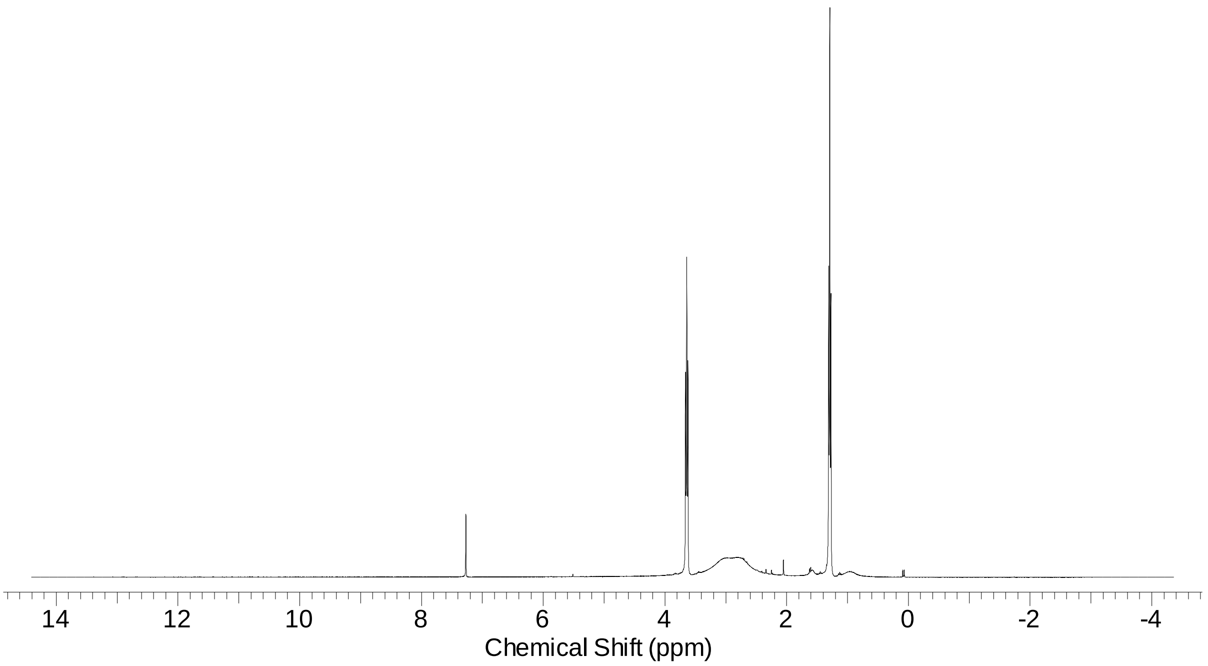

Figure S17 ^{1}H NMR spectrum after 173 hrs of: CEES (0.20 mM), $Cu(acac)_2$ (0.01 mM) and $CDCl_3$ (1.00 mL).

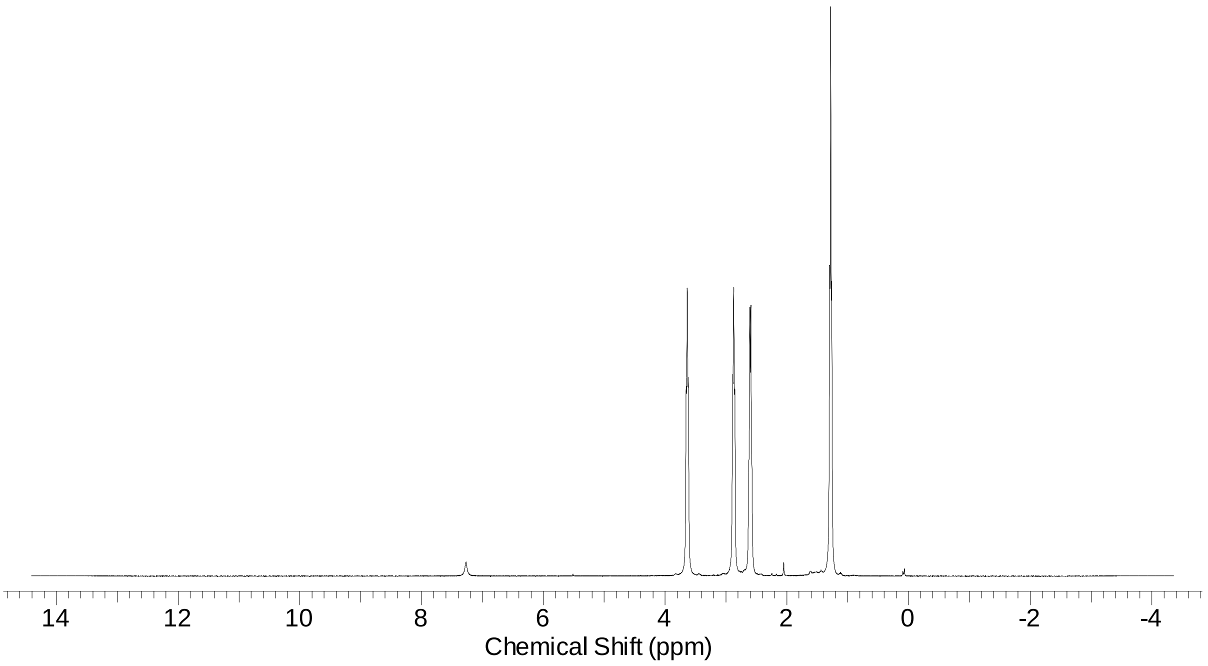

Figure S18 ^{1}H NMR spectrum after 173 hrs of: CEES (0.20 mM), $Fe(acac)_3$ (0.01 mM) and $CDCl_3$ (1.00 mL).

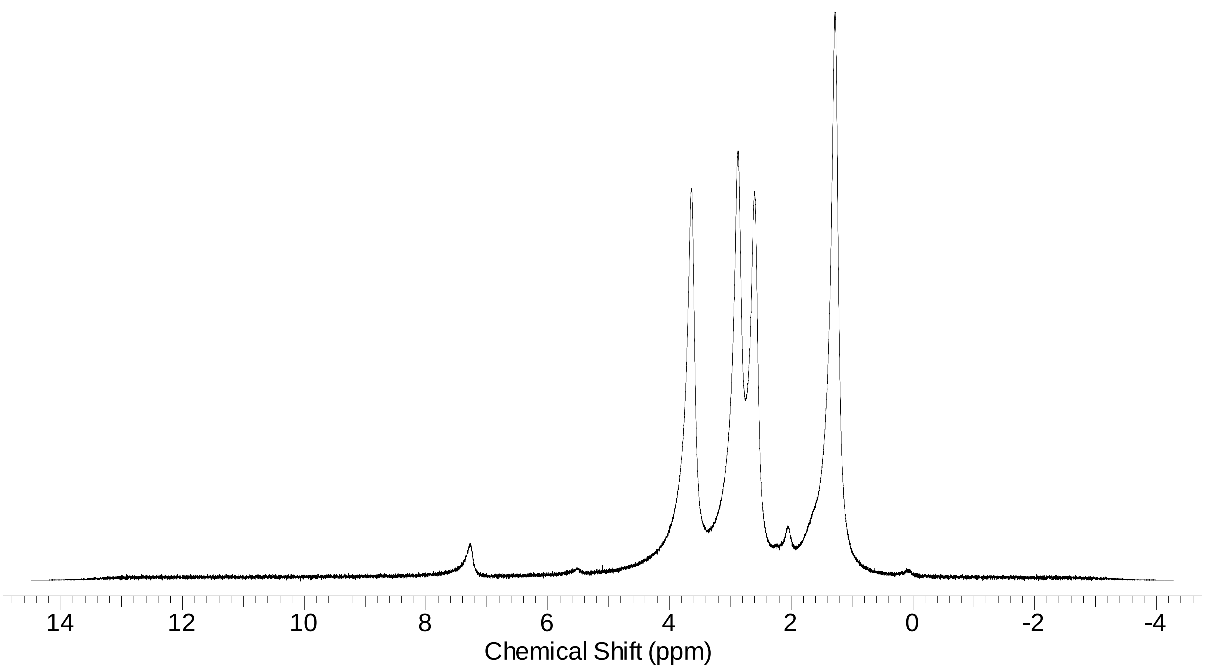

Figure S19 ^{1}H NMR spectrum after 173 hrs of: CEES (0.20 mM), Mn(acac)$_3$ (0.01 mM) and $CDCl_3$ (1.00 mL).

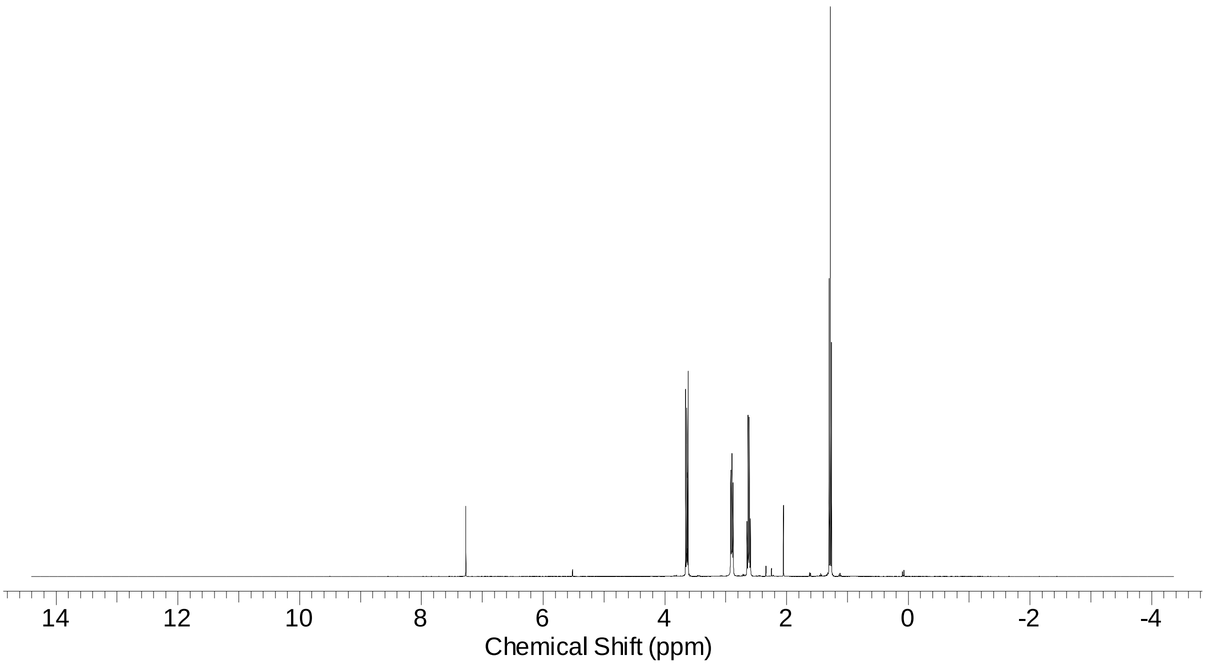

Figure S20 ^{1}H NMR spectrum after 173 hrs of: CEES (0.20 mM), Ni(acac)$_2$ (0.01 mM) and $CDCl_3$ (1.00 mL).

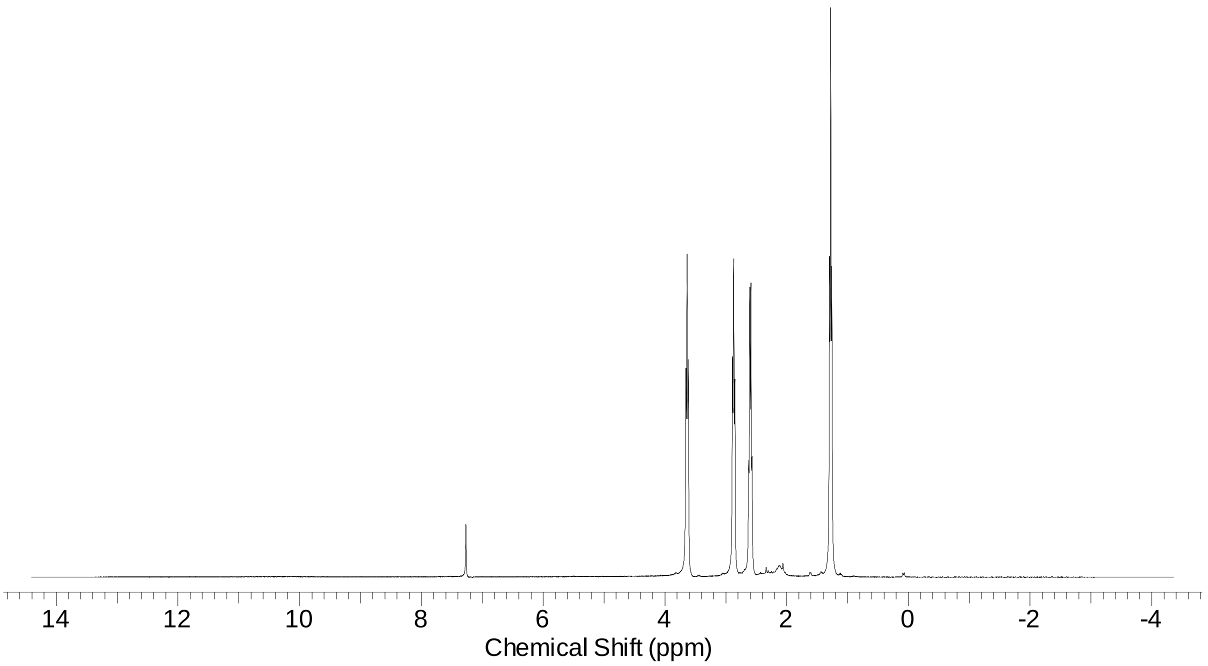

Figure S21 ^{1}H NMR spectrum after 173 hrs of: CEES (0.20 mM), $VO(acac)_3$ (0.01 mM) and $CDCl_3$ (1.00 mL).

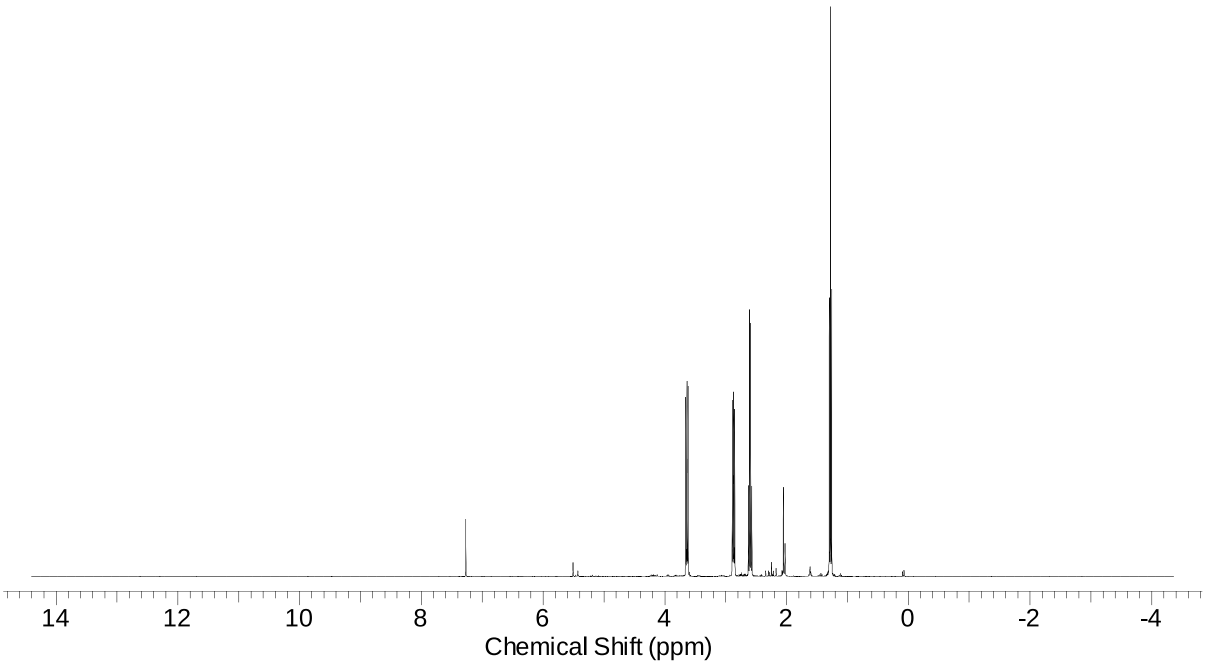

Figure S22 ^{1}H NMR spectrum after 173 hrs of: CEES (0.20 mM), $Zn(acac)_2$ (0.01 mM) and $CDCl_3$ (1.00 mL).

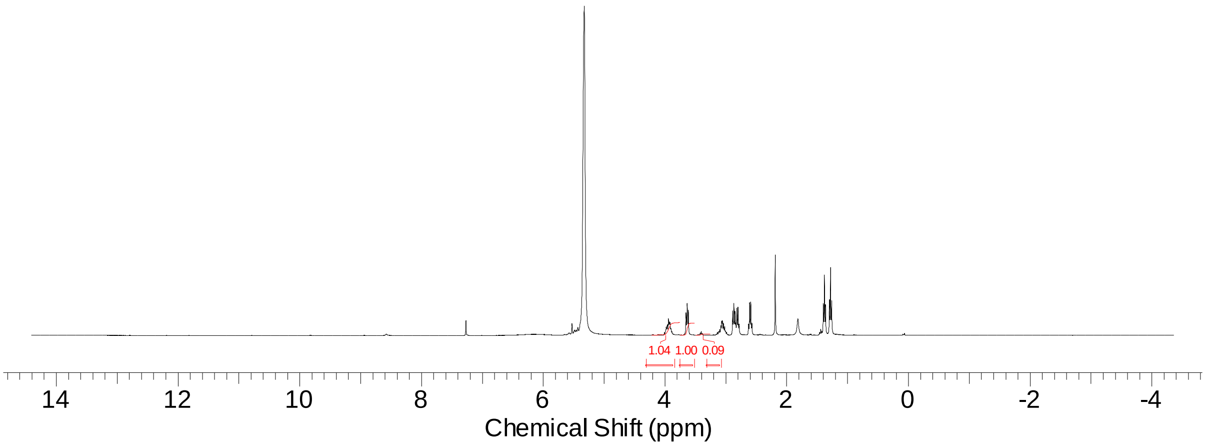

Figure S23 ^{1}H NMR spectrum after 165 hrs of: CEES (0.20 mM), $Co(acac)_3$ (0.01 mM), $CDCl_3$ (1.00 mL) and 30% hydrogen peroxide in water (0.10 mL).

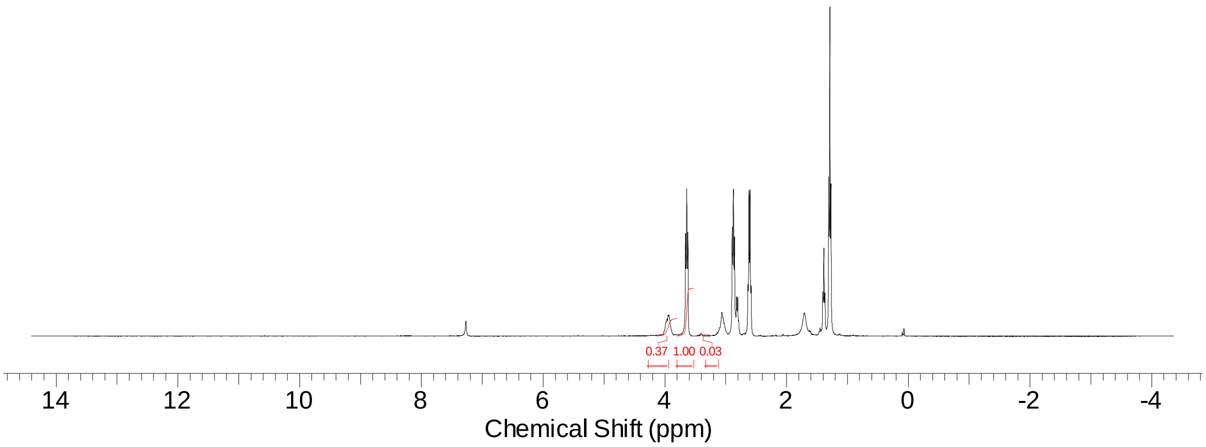

Figure S24 ^{1}H NMR spectrum after 165 hrs of: CEES (0.20 mM), $Cr(acac)_3$ (0.01 mM), $CDCl_3$ (1.00 mL) and 30% hydrogen peroxide in water (0.10 mL).

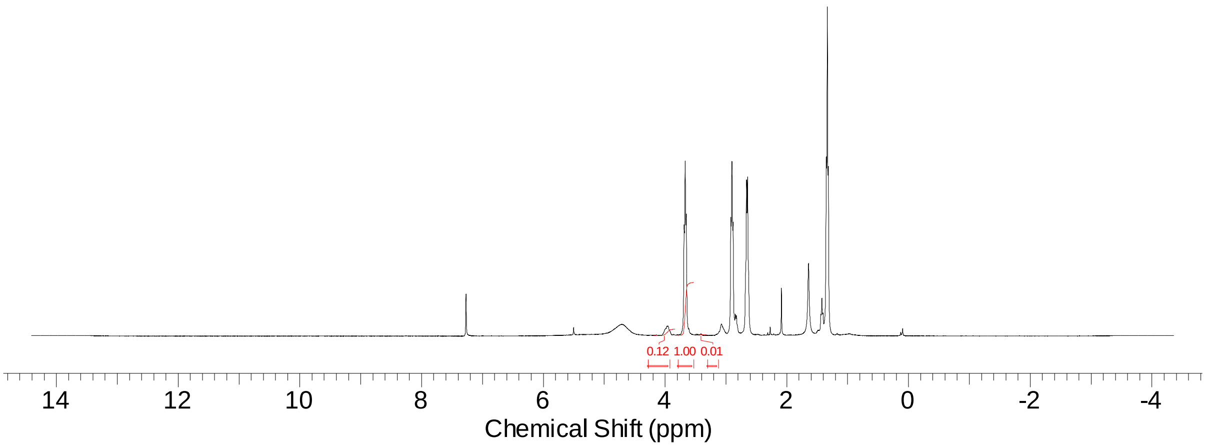

Figure S25 ^{1}H NMR spectrum after 165 hrs of: CEES (0.20 mM), $Cu(acac)_2$ (0.01 mM), $CDCl_3$ (1.00 mL) and 30% hydrogen peroxide in water (0.10 mL).

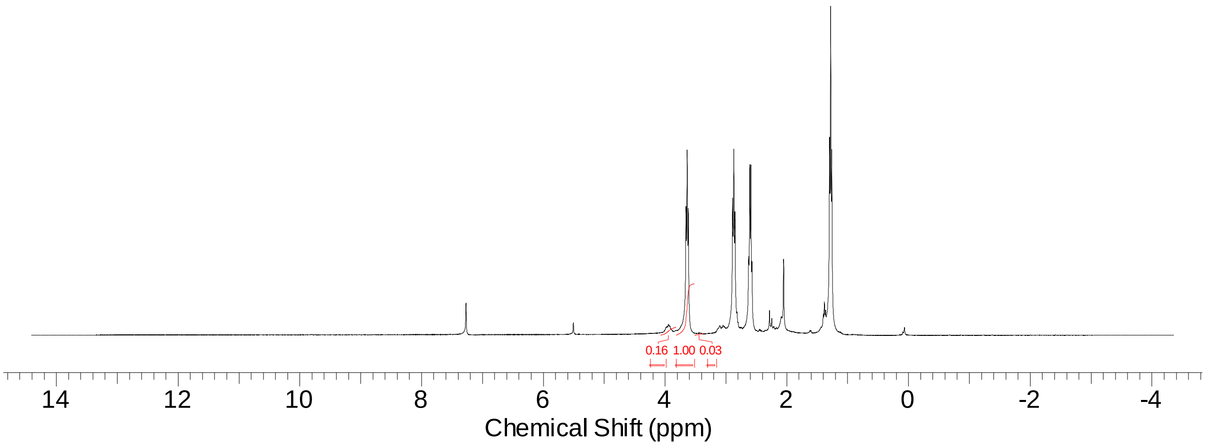

Figure S26 ^{1}H NMR spectrum after 165 hrs of: CEES (0.20 mM), $Fe(acac)_3$ (0.01 mM), $CDCl_3$ (1.00 mL) and 30% hydrogen peroxide in water (0.10 mL).

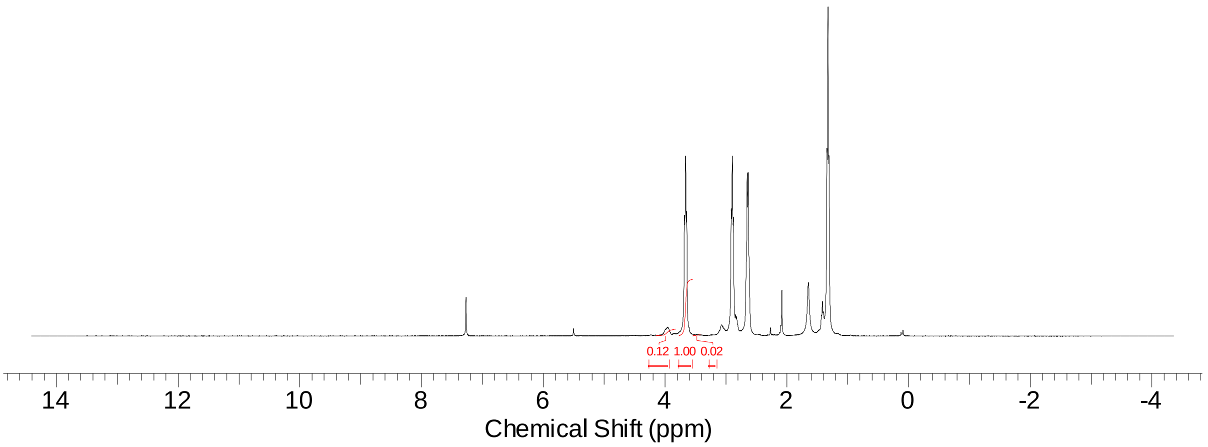

Figure S27 ^{1}H NMR spectrum after 165 hrs of: CEES (0.20 mM), $Ni(acac)_2$ (0.01 mM), $CDCl_3$ (1.00 mL) and 30% hydrogen peroxide in water (0.10 mL).

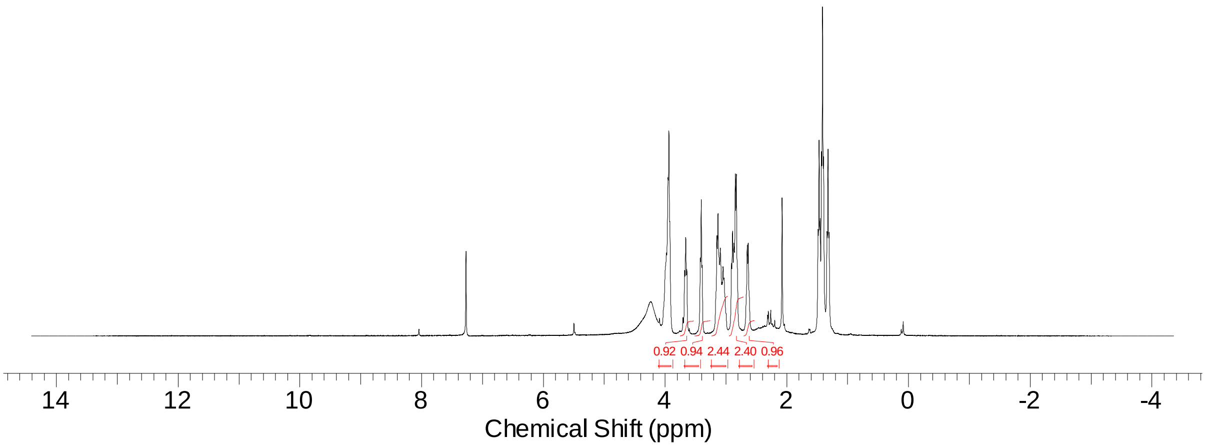

Figure S28 ^{1}H NMR spectrum after 165 hrs of: CEES (0.20 mM), $VO(acac)_2$ (0.01 mM), $CDCl_3$ (1.00 mL) and 30% hydrogen peroxide in water (0.10 mL).

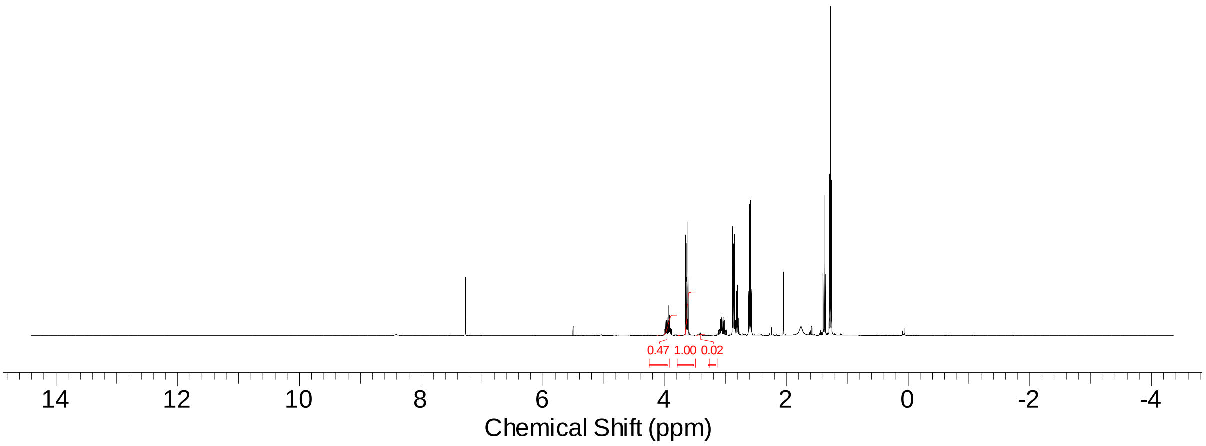

Figure S29 ^{1}H NMR spectrum after 165 hrs of: CEES (0.20 mM), $Zn(acac)_2$ (0.01 mM), $CDCl_3$ (1.00 mL) and 30% hydrogen peroxide in water (0.10 mL).

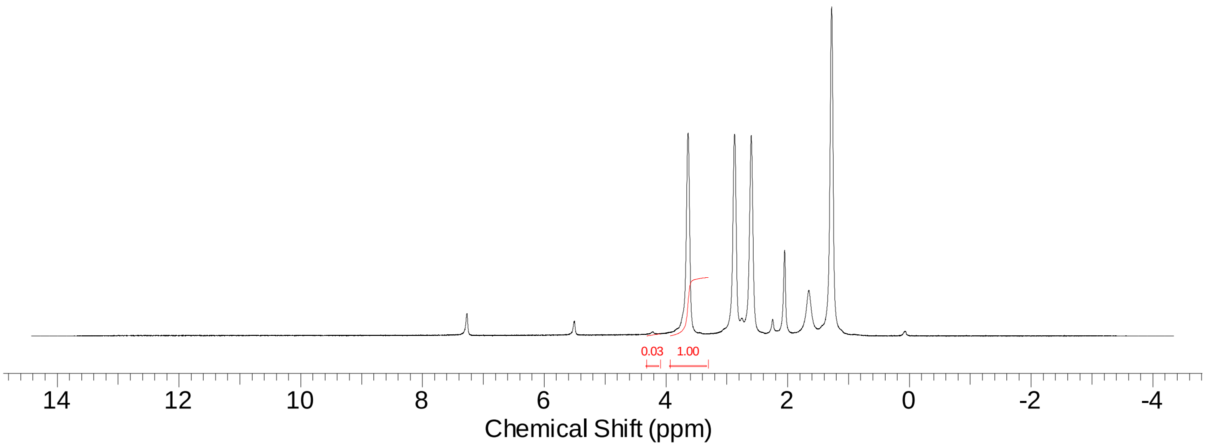

Figure S30 ^{1}H NMR spectrum after 147 hrs of: CEES (0.20 mM), $Mn(acac)_3$ (0.01 mM), $CDCl_3$ (1.00 mL) and 30% hydrogen peroxide in water (0.10 mL).

Secondary Solution state studies

Experimental Method: Samples were prepared, as detailed in Table S3, in a single NMR tube per experiment, with the hydrogen peroxide layered on top of the $CDCl_3$ mixture containing catalyst and/or simulant as appropriate. The addition of the peroxide was taken as time = 0, at this point it was assumed that no oxidation products were present as ^{1}H NMR of the stock CEES in $CDCl_3$ showed no breakdown products to be present. The samples were then sealed and briefly shaken by hand for approximately two seconds before being allowed to separate. The samples were disturbed as little as possible for the course of the experiment with the temperature of the samples maintained at approximately 18 ± 2 °C. All experiments were monitored by ^{1}H NMR. The percentage conversion of simulant to oxidised species was calculated through comparative integration of simulant and product peaks, the results of which are given in Table S4. The initial and final ^{1}H NMR spectra for each experiment are shown in Figures S31-34.

Table S4 Constituents of samples 21-23. [a] – 30 wt% solution in water.

Sample number	Catalyst	Conc. (mM)	Solvent	Amount (mL)	Simulant	Conc. (mM)	Peroxide	Amount (mL)
21	-	-	$CDCl_3$	1.00	CEES	0.20	Hydrogen peroxide[a]	0.1
22	$Cu(acac)_2$	0.01	$CDCl_3$	1.00	CEES	0.20	Hydrogen peroxide[a]	0.1
23	$Cu(hfac)_2.H_2O$	0.01	$CDCl_3$	1.00	CEES	0.20	Hydrogen peroxide[a]	0.1

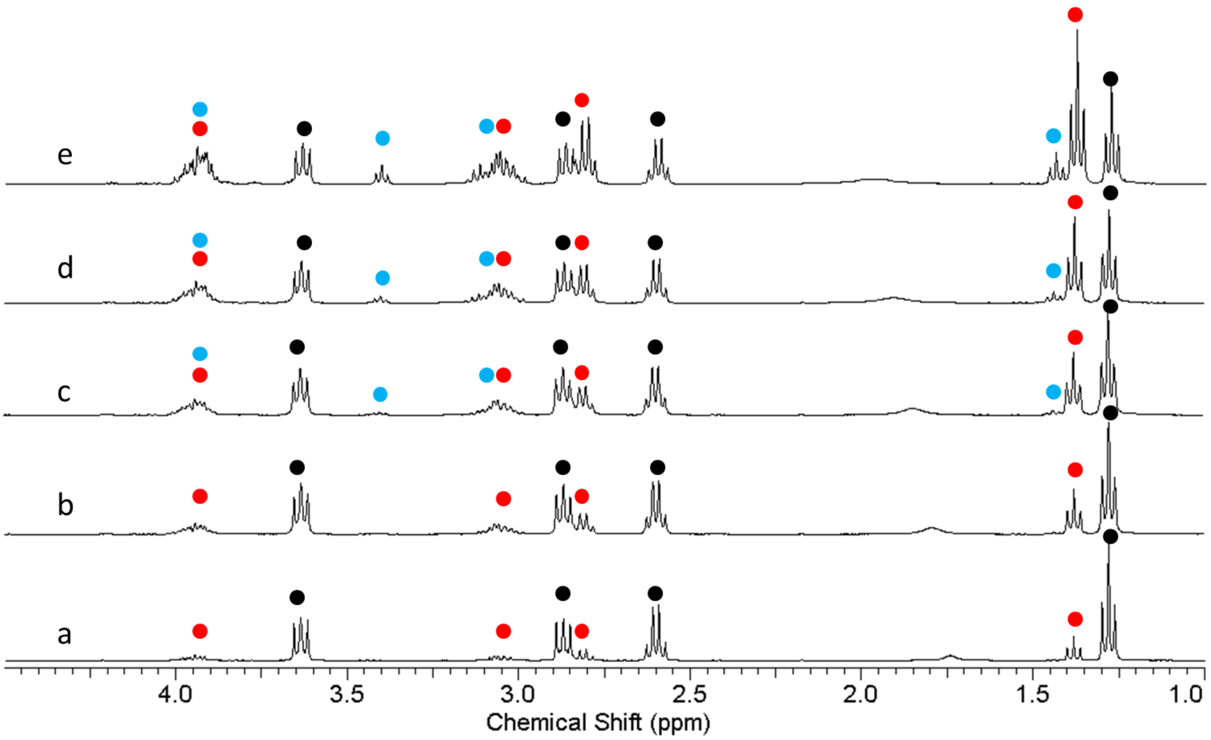

Figure S31 ^{1}H NMR spectrum of CEES (0.20 mM), $Cu(hfac)_2.H_2O$ (0.01 mM), $CDCl_3$ (1.00 mL) and 30% hydrogen peroxide in water (0.10 mL): **black** - CEES; **red** - sulfoxide; **blue** - sulfone. a) T = 19.48 hrs; b) T = 29.08 hrs; c) T = 53.48 hrs; d) T = 81.13 hrs and e) T = 124.22 hrs.

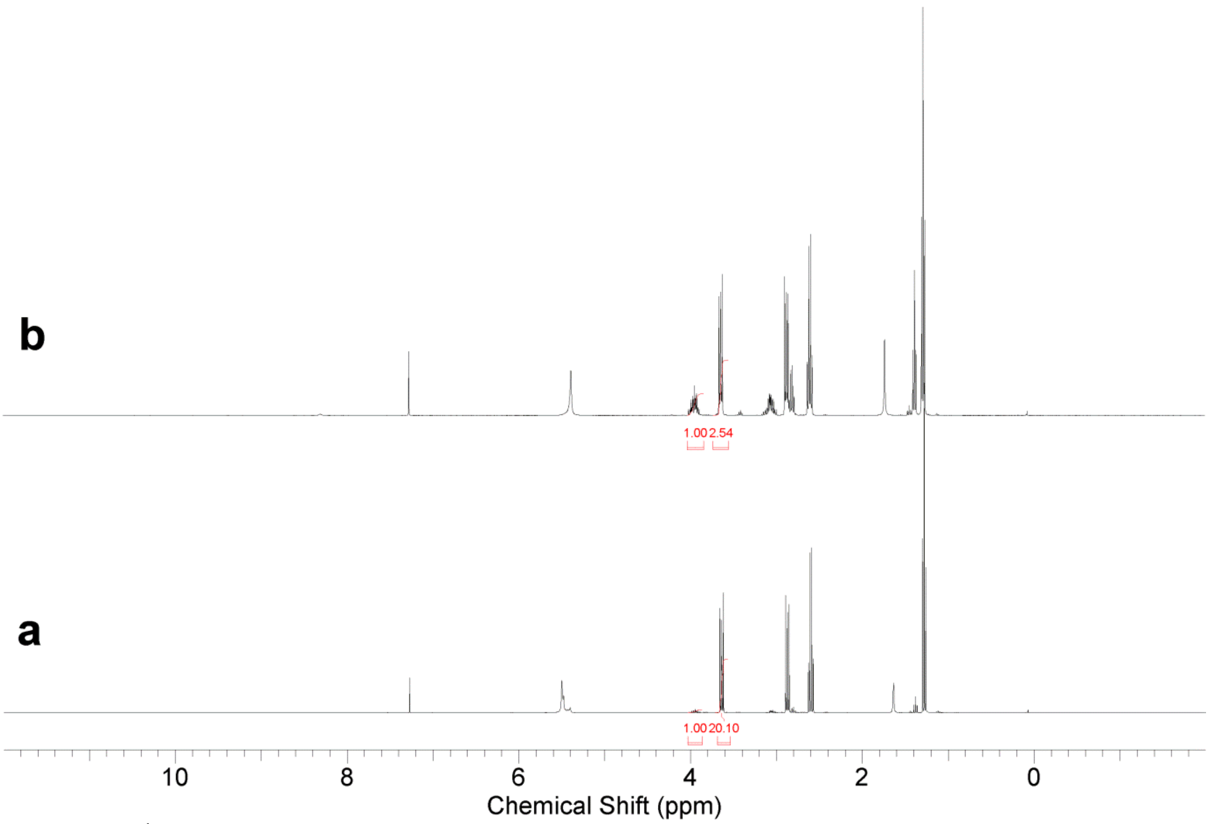

Figure S32: ^{1}H NMR spectra of sample **21** a) time = 20 hours; b) 124 hours.

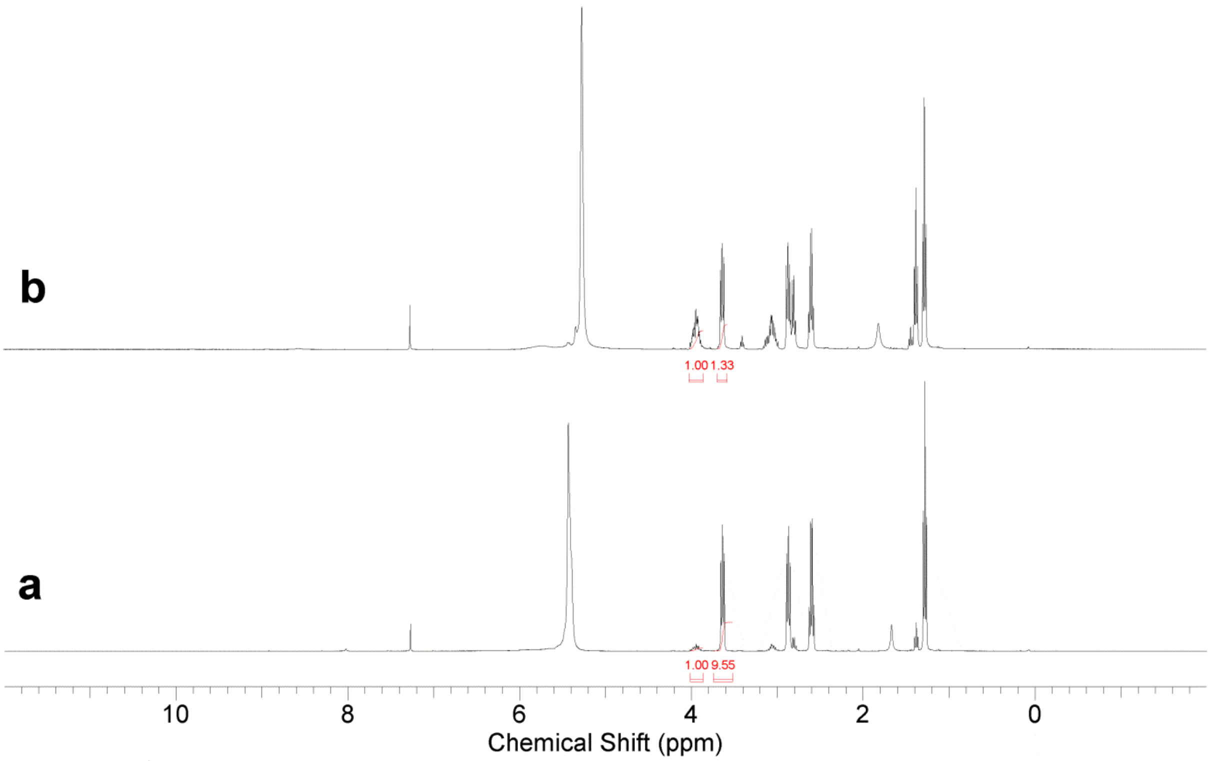

Figure S33: ^{1}H NMR spectra of sample **22** a) time = 19.48 hours; b) 124.21.

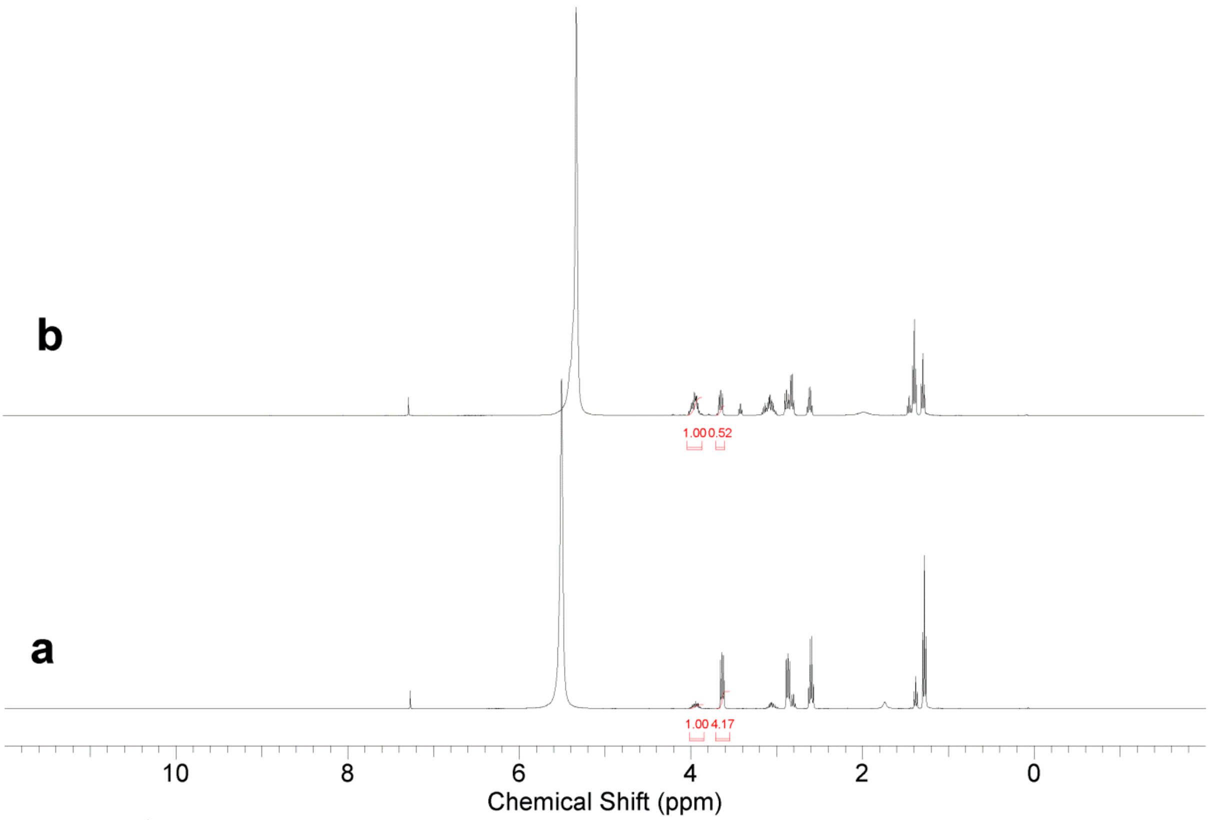

Figure S34: ^{1}H NMR spectra of sample **23** a) time = 19.40 hours; b) 124.00.

Solid state studies

Experimental Method: Samples were prepared, as detailed in Table S5, in a single NMR tube per experiment. Those sample containing compound **1** to gelate the sample were briefly heated for 15 seconds to initially dissolve the gelator so that the gel could from upon cooling. The addition of the final component of the mixture was taken as time = 0; at this point it was assumed that no oxidation products were present as ^{1}H NMR of the stock CEES in $CDCl_3$ showed no breakdown products to be present. The samples were then sealed and briefly shaken by hand for approximately two seconds to ensure the even distribution of components within the solution. The samples were disturbed as little as possible for the course of the experiment with the temperature of the samples maintained at 18 ± 2 °C. All experiments were monitored by ^{1}H NMR. The percentage conversion of simulant to oxidised species was calculated through comparative integration of simulant and product peaks, the results of which are given in Table S6. The initial and final ^{1}H NMR spectra for each experiment are shown in Figures S35-S37.

Table S5 Constituents of samples 5-7. [a] – supplied in a 5-6 M solution in decane.

Sample number	Catalyst	Conc. (mM)	Solvent	Amount (mL)	Simulant	Conc. (mL)/(mM)	Peroxide	Amount (mL)/(mM)	Gelator	Amount (mg/mL)
24	-	-	$CDCl_3$	0.921	CEES	0.029/0.25	*t*-butyl peroxide[a]	0.05/0.25-0.30	**1**	40
25	$Cu(hfac)_2.H_2O$	0.01	$CDCl_3$	0.921	CEES	0.029/0.25	*t*-butyl peroxide[a]	0.05/0.25-0.30	**1**	40
26	$Cu(hfac)_2.H_2O$	0.04	$CDCl_3$	0.684	CEES	0.116/1.00	*t*-butyl peroxide[a]	0.2/1.00-1.20	**2**	24

Table S6 Percentage of CEES oxidised over time. *a* – an estimation of starting material conversion could not be made due to the composition of the sample at this time point prevented the NMR machine locking to the sample. *b* – overlapping and peak broadening prevents accurate integration of the NMR spectra. *c* – no CEES present to be oxidised, sample remained stable.

Sample Number										
	24	**Time (Hrs)**	0	0.37	18.37	20.27	26.72	42.80	69.03	153.90
		**% conversion**	0	0	9.56	*a*	12.72	*a*	28.8	48.87
	25	**Time (Hrs)**	0	0.27	18.25	20.48	26.62	> 26.62		
		**% conversion**	0	*b*	a	a	50	*a*		
	26	**Time (Hrs)**	0	1.92	10.88	23.42	30.83	46.95	74.10	158.10
		**% conversion**	0	*b*	*b*	*b*	*b*	*b*	*b*	*b*

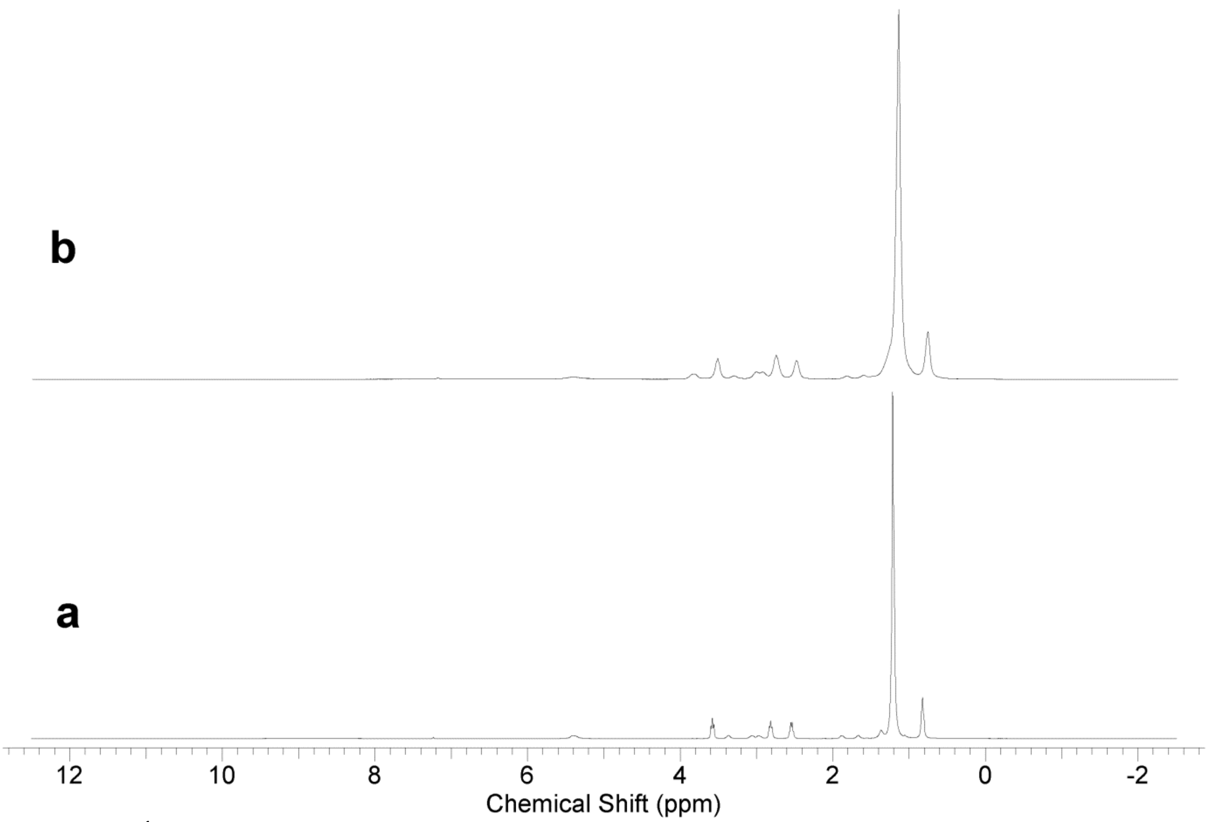

Figure S35: ^{1}H NMR spectra of sample 24 a) time = 0.37 hrs; b) 26.72 hrs.

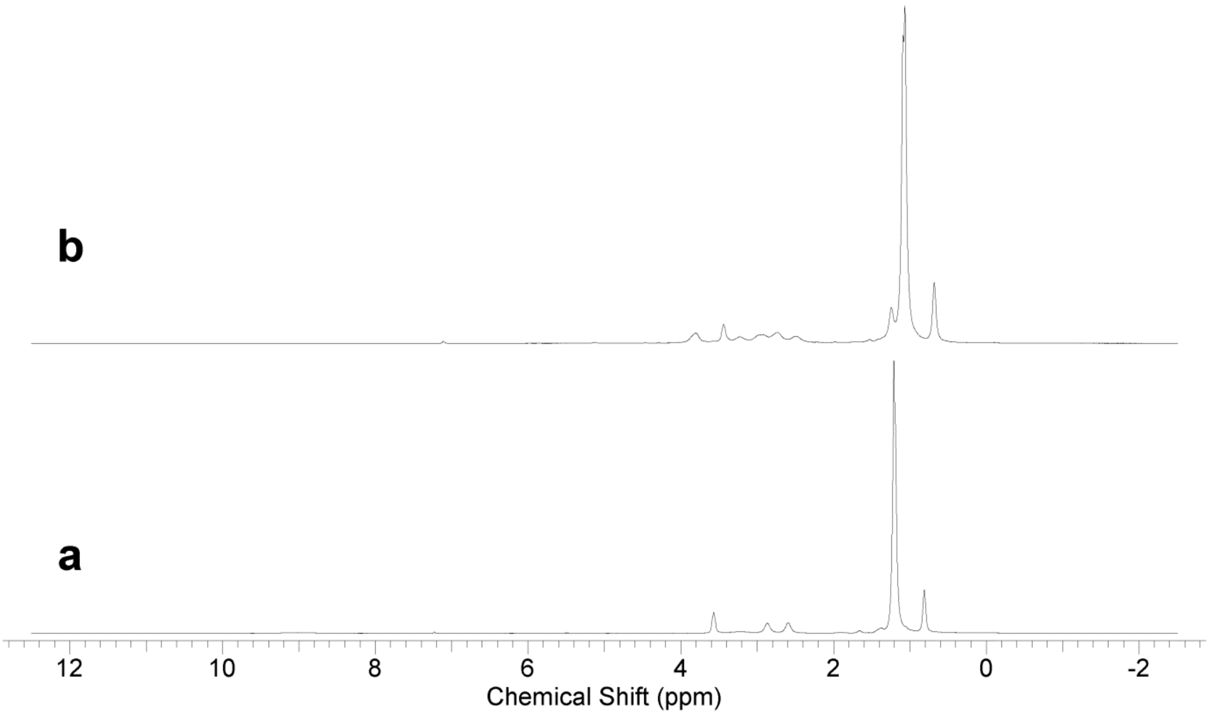

Figure S36: ^{1}H NMR spectra of sample 25 a) time = 0.27 hrs; b) 26.62 hrs.

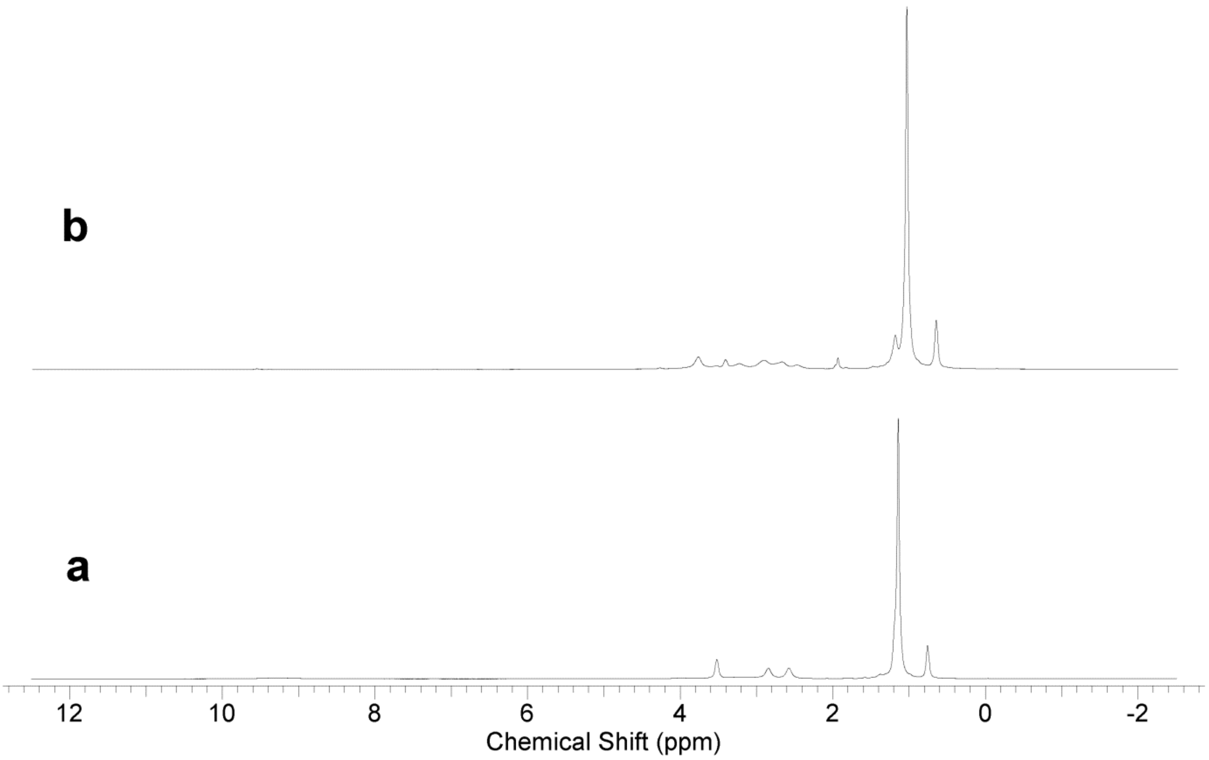

Figure S37: ^{1}H NMR spectra of sample 26 a) time = 1.92 hrs; b) 158.10 hrs.

Gel formation

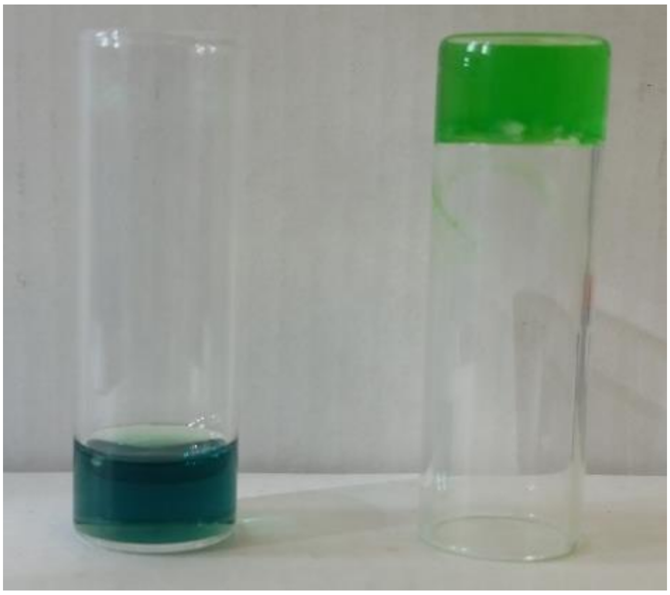

Figure S38 left to right 5 mg vs 45 mg of **1** in 1 ml of a $Cu(hfac)_2.H_2O$ saturated chloroform solution, illustrating colour change and evidence of competitive Cu complexation.

Sol and Gelator simulant studies

Experimental Method: Samples were prepared, as detailed in Table S7, in a single NMR tube per experiment. Those samples containing compound **1** were briefly heated for 15 seconds to initially dissolve the gelator. The addition of the final component of the mixture was taken as time = 0. At this point it was assumed that no oxidation products were present as ^1H NMR of the stock CEES in $CDCl_3$ showed no breakdown products to be present. The samples were then sealed and briefly shaken by hand for approximately two seconds to ensure the even distribution of components within the solution. The samples were disturbed as little as possible for the course of the experiment with the temperature of the samples maintained at approximately 18 ± 2 °C. All experiments were monitored by ^1H NMR. The percentage conversion of simulant to oxidised species was calculated through comparative integration of simulant and product peaks, the results of which are given in Table S8. The initial and final ^1H NMR spectra for each experiment are shown in Figures S39-S41.

Although a gel was found to form under these conditions in the presence of **1** (sample 27), addition of $Cu(hfac)_2.H_2O$ (sample 28) prevented gel formation meaning this sample remained a sol. We believe that this is due to the interactions of the gelator with the Cu(II) complex altering material formation processes. These spectra suggest that under these reaction conditions the CEES undergoes primary oxidation to the sulfoxide. Secondary oxidation of sulfoxide to the sulfone was not observed by these analysis methods.

Table S7 Constituents of samples 27-29 supplied. The t-butyl peroxide was supplied to each sample in a 5-6 M decane solution.

Sample number	catalyst	Conc. (mM)	Solvent	Amount (mL)	simulant	Conc. (mL)/(mM)	Gel formed	Peroxide (mL)/(mM)	Gelator	Amount (mg/mL)
**27**	-	-	$CDCl_3$	0.684	CEES	0.116/1.00	yes	0.2/1.00-1.20	**1**	30
**28**	Cu(hfac)$_2$.H_2O	0.04	$CDCl_3$	0.684	CEES	0.116/1.00	no	0.2/1.00-1.20	**1**	30
**29**	Cu(hfac)$_2$.H_2O	0.04	$CDCl_3$	0.684	CEES	0.116/1.00	no	0.2/1.00-1.20	**2**	24

Table S8 Percentage of CEES to undergo primary oxidation. *a* – overlapping and peak broadening prevents accurate integration of the NMR spectra.

**Sample Number**										
	**8**	**Time (Hrs)**	0	1.55	10.53	23.22	30.47	46.60	72.92	169.2
		**% conversion**	0	1.42	22.40	32.40	38.16	44.23	51.37	66.41
	**10**	**Time (Hrs)**	0	1.82	10.80	23.33	30.73	46.87	73.18	158.00
		**% conversion**	0	4.69	27.09	43.37	47.39	52.92	55.09	66.02
	**14**	**Time (Hrs)**	0	1.92	10.88	23.42	30.83	46.95	74.10	158.10
		**% conversion**	0	*a*	*a*	*a*	*a*	*a*	*a*	*a*

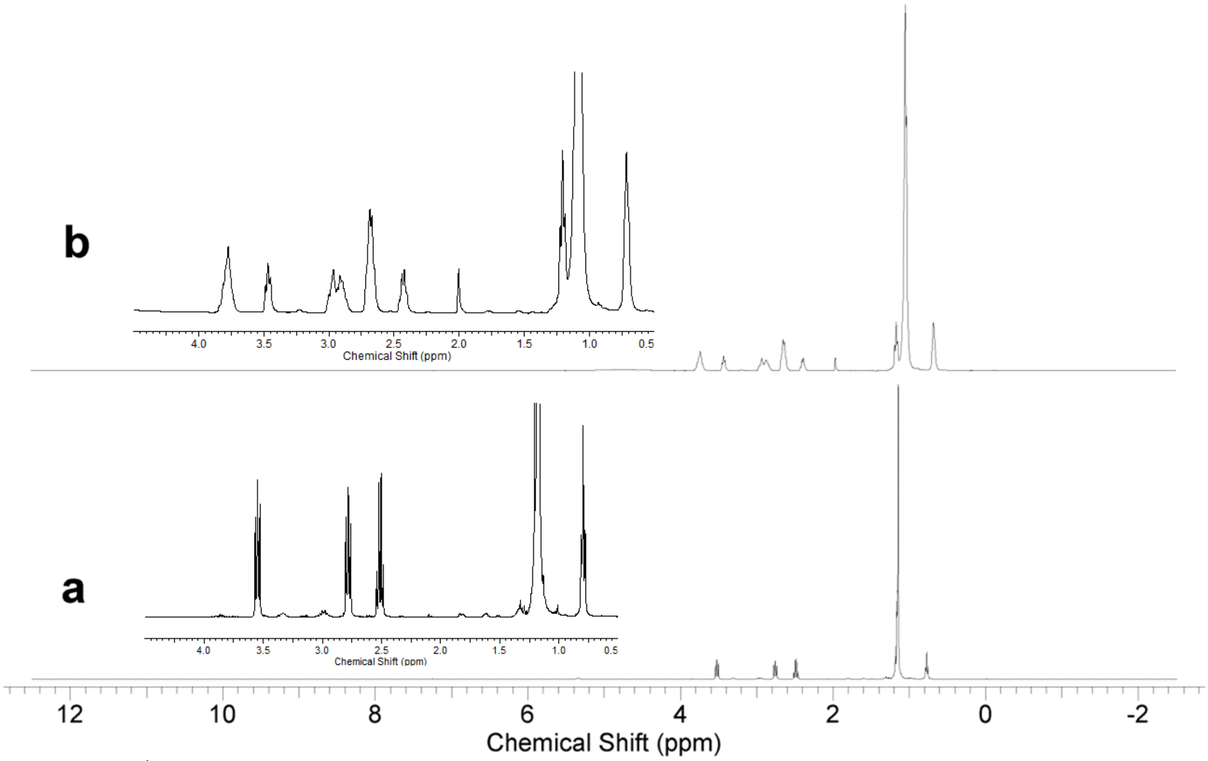

Figure S39: ^{1}H NMR spectra of sample 27 a) time = 1.55 hrs; b) 169.20 hrs.

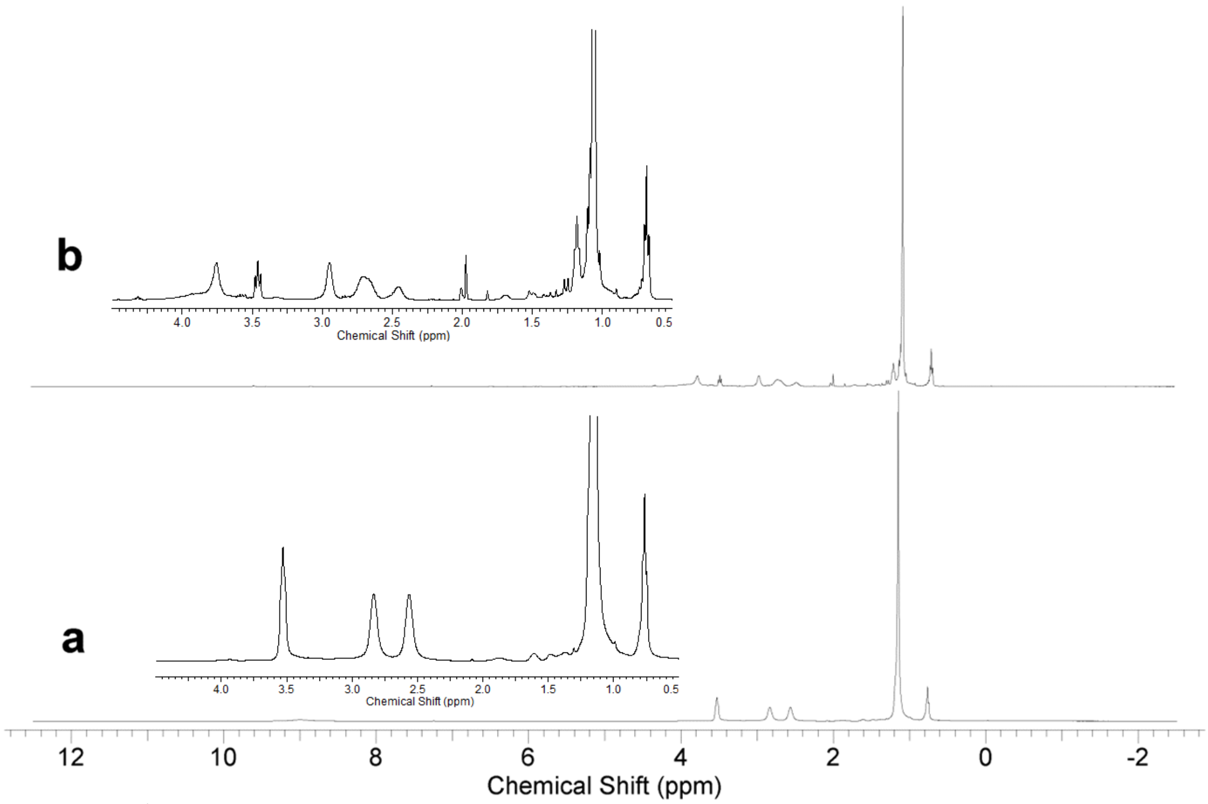

Figure S40: ^{1}H NMR spectra of sample 28 a) time = 1.82 hrs; b) 158.00 hrs.

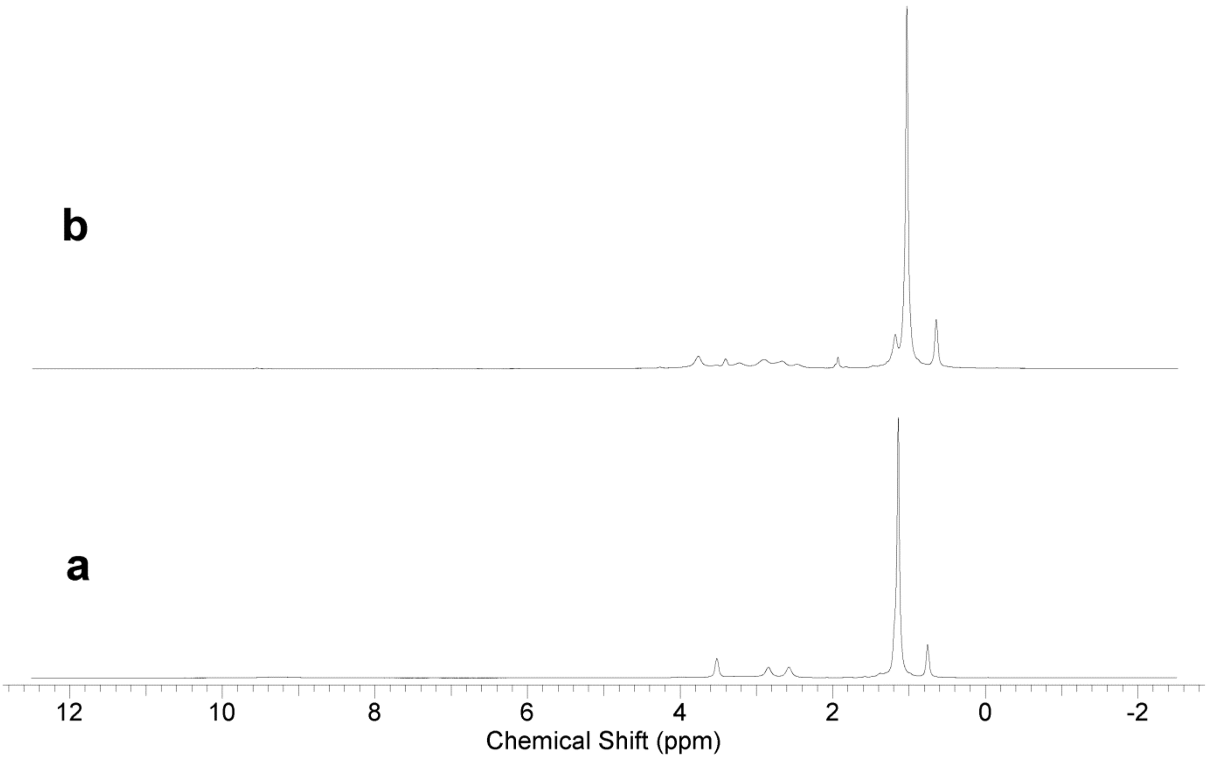

Figure S41: ^{1}H NMR spectra of sample 29 a) time = 1.92 hrs; b) 158.10 hrs.

References

1. S. Stoll and A. Schweiger, *J. Magn. Reson.* , 2006, **178**, 42-55.
2. A. W. Maverick, F. R. Fronczek, E. F. Maverick, D. R. Billodeaux, Z. T. Cygan and R. A. Isovitsch, *Inorg. Chem.*, 2002, **41**, 6488-6492.
3. C. Glidewell;, *Inorganic Experiments 2nd Ed*, Wiley and Sons, 2006.
4. J. van Esch, F. Schoonbeek, M. de Loos, H. Kooijman, A. L. Spek, R. M. Kellogg and B. L. Feringa, *Chem. Eur. J.*, 1999, **5**, 937-950.
5. E. M. N. AbdelHafez, O. M. Aly, G. Abuo-Rahma and S. B. King, *Adv. Syn. Catal.*, 2014, **356**, 3456-3464.
6. M. F. Farona, D. C. Perry and H. A. Kuska, *Inorg. Chem.*, 1968, **7**, 2415-2418.
7. H. Yokoi, *Inorg. Chem.*, 1978, **17**, 538-542.
8. P. C. B. Page, B. R. Buckley, C. Elliott, Y. Chan, N. Dreyfus and F. Marken, *Synlett*, 2016, **27**, 80-82.
9. Y. Y. Liu, A. J. Howarth, J. T. Hupp and O. K. Farha, *Angew. Chem. Int. Edit.*, 2015, **54**, 9001-9005.
